# Hematopoietic cell– versus enterocyte-derived dipeptidyl peptidase-4 differentially regulates triglyceride excursion in mice

**DOI:** 10.1172/jci.insight.140418

**Published:** 2020-08-20

**Authors:** Elodie M. Varin, Antonio A. Hanson, Jacqueline L. Beaudry, My-Anh Nguyen, Xiemin Cao, Laurie L. Baggio, Erin E. Mulvihill, Daniel J. Drucker

**Affiliations:** 1 Lunenfeld-Tanenbaum Research Institute, Department of Medicine, Mt. Sinai Hospital, Toronto, Ontario, Canada.; 2 Department of Biochemistry, Microbiology and Immunology, University of Ottawa, Ottawa, Ontario, Canada.; 3 University of Ottawa Heart Institute, Ottawa, Ontario, Canada.

**Keywords:** Endocrinology, Diabetes

## Abstract

Postprandial triglycerides (TGs) are elevated in people with type 2 diabetes (T2D). Glucose-lowering agents, such as glucagon-like peptide-1 (GLP-1) receptor agonists and dipeptidyl peptidase-4 (DPP-4) inhibitors, also reduce postprandial TG excursion. Although the glucose-lowering mechanisms of DPP-4 have been extensively studied, how the reduction of DPP-4 activity improves lipid tolerance remains unclear. Here, we demonstrate that gut-selective and systemic inhibition of DPP-4 activity reduces postprandial TG excursion in young mice. Genetic inactivation of *Dpp4* simultaneously within endothelial cells and hematopoietic cells using *Tie2*-Cre reduced intestinal lipoprotein secretion under regular chow diet conditions. Bone marrow transplantation revealed a key role for hematopoietic cells in modulation of lipid responses arising from genetic reduction of DPP-4 activity. Unexpectedly, deletion of *Dpp4* in enterocytes increased TG excursion in high-fat diet–fed (HFD-fed) mice. Moreover, chemical reduction of DPP-4 activity and increased levels of GLP-1 were uncoupled from TG excursion in older or HFD-fed mice, yet lipid tolerance remained improved in older *Dpp4*^–/–^ and *Dpp4*^EC–/–^ mice. Taken together, this study defines roles for specific DPP-4 compartments, age, and diet as modifiers of DPP-4 activity linked to control of gut lipid metabolism.

## Abstract 2

Gut-selective and systemic inhibition of dipeptidyl peptidase-4 activity reveals roles in gut lipid metabolism.

## Graphical abstract

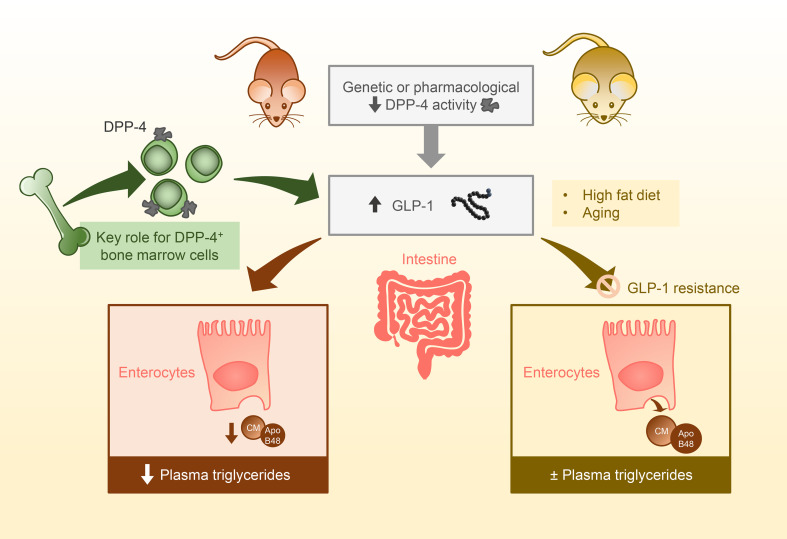

## Introduction

Elevated concentrations of postprandial triglyceride-rich lipoproteins (TRLs) are associated with an increased incidence of cardiovascular disease (1), and therapies that lower triglycerides (TGs) have demonstrated cardiovascular benefit (2). TRLs, including very-low-density lipoproteins and chylomicrons, are elevated in the setting of insulin resistance and type 2 diabetes (T2D), reflecting impaired clearance and dysregulated TG secretion from both the liver and intestine (3). These findings have translational relevance, as elevated levels of plasma TGs may contribute to the excess burden of cardiovascular risk in people with T2D (4).

Among the various glucose-lowering agents used to treat T2D, 2 classes of incretin-based therapies, glucagon-like peptide-1 (GLP-1) receptor agonists (GLP-1RA) and dipeptidyl peptidase-4 (DPP-4) inhibitors (DPP4i), lower postprandial TGs (5, 6). The metabolic actions of GLP-1 to control insulin, glucagon, glucose, and body weight are mediated by a single widely expressed GLP-1R (7, 8). Moreover, the acute lipid-lowering actions of GLP-1RA are independent of the control of islet hormone secretion or gastric emptying and inhibited by the GLP-1R antagonist exendin (9–39), consistent with a key role for the canonical GLP-1R in the control of postprandial TG secretion (9, 10).

DPP4i lower glucose in part through activation of the GLP-1R in mice and humans (11–13). These drugs also lower postprandial TGs and apoB48 in humans (5, 14, 15) and in mice through mechanisms requiring the GLP-1R (9). Interestingly, intracerebroventricular administration of a DPP4i produces an acute TG-lowering effect in hamsters (16). Nevertheless, the mechanisms by which peripherally administered DPP4i inhibits enterocyte chylomicron secretion have not been determined.

DPP-4 is a ubiquitous cell membrane–associated serine protease expressed in many tissues, including the gut (17, 18); it also circulates as a catalytically active soluble form. Here, we studied the cellular sites of DPP-4 expression required for control of lipid metabolism in mice using gut-selective versus systemic inhibitory concentrations of the DPP4i sitagliptin (19, 20). We also determined the relative importance of *Dpp4* expression within endothelial, hematopoietic, and gut epithelial cells for control of lipid metabolism in mice fed regular chow (RC) or high-fat diets (HFD). Finally, we assessed the relative importance of *Tie2*-sensitive versus *Vil1*-Cre–targeted DPP-4^+^ cell populations for transducing the lipid-lowering actions of gut-selective versus systemically effective doses of DPP4i in mice under RC and HFD conditions.

## Results

### Systemic and gut-selective inhibition of DPP-4 activity improves lipid tolerance and reduces TG production in young RC-fed mice.

**Figure 1:**
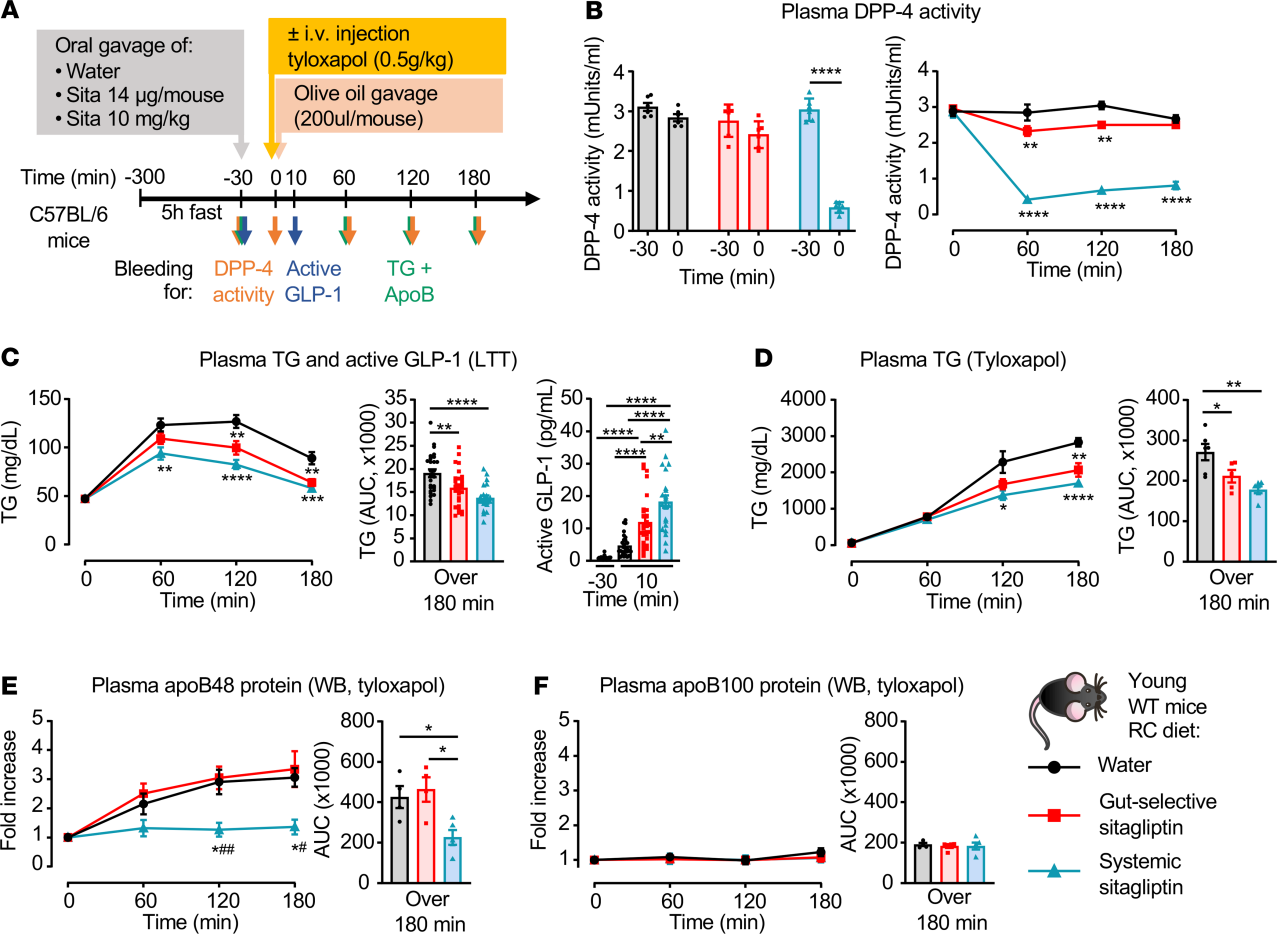
Sitagliptin improves lipid tolerance and TG production in young RC-fed mice. (**A**) Experimental model used for data shown in **B–F**. 8- to 12-week-old regular chow–fed (RC-fed) WT mice were fasted for 5–6 hours and then given oral administration of water or gut-selective (14 μg/mouse) or systemic (10 mg/kg) doses of sitagliptin at time –30 minutes. Mice were then gavaged with 200 μl olive oil only (for lipid tolerance [LTT] in **B** and **C**) or given an i.v. injection of 0.5 g/kg tyloxapol just before olive oil (for TG and ApoB production in **D**–**F**). (**B** and **C**) Plasma DPP-4 activity just before (time –30 minutes), and at 30 (time 0), 60, 120, and 180 minutes after oral gavage or water of sitagliptin, as indicated (**B**, *n* = 6–8/group). (**C**) Plasma TG and AUC over 3 hours (left and middle) and plasma active GLP-1 levels 30 minutes before and 10 minutes after oral gavage of olive oil (right, *n* = 23–29/group) during a LTT. (**D**–**F**) Plasma TG and AUC over 180 minutes (**D**), and apoB48 and apoB100 protein levels measured by Western blot (WB) (**E** and **F**, *n* = 6–8/group) after oral gavage of water or sitagliptin, followed by i.v. injection of tyloxapol and oral gavage of olive oil, as described in **A**. Data are presented as mean ± SEM. Each *n* represents a biological replicate from 4 independent cohorts (**C**) and 1 group (**B** and **D**–**F**) of sex- and age-matched animals. (**B**–**D**) *P < 0.05, **P < 0.01, ***P < 0.001, ****P < 0.0001, using 1-way ANOVA with Tukey’s correction for multiple comparisons for indicated groups. (**E**) *P < 0.05 vs. water, ^#^*P* < 0.05 and ^##^*P* < 0.01 vs. gut-selective dose of sitagliptin using 1-way ANOVA with Tukey’s correction for multiple comparisons for indicated groups.

Studies of glucose metabolism have shown that low doses of DPP4i that reduce DPP-4 activity within the gut, but not meaningfully in the systemic circulation, are sufficient to improve glucose tolerance (19, 20). Accordingly, we used low and high doses of sitagliptin, with or without tyloxapol, to assess how acute DPP-4 inhibition modulates lipid tolerance in mice (Figure 1A). Consistent with our previous findings (20), oral gavage of systemically active doses of sitagliptin (10 mg/kg) inhibited plasma DPP-4 activity by approximately 85% when assessed 30 minutes after oral administration (Figure 1B). DPP-4 activity remained suppressed at 210 minutes after gavage of sitagliptin (180 minutes after olive oil gavage) (Figure 1B). In contrast, plasma DPP-4 activity was modestly and transiently reduced by lower doses (14 μg/mouse) of sitagliptin (Figure 1B), shown to selectively target DPP-4 activity within the intestinal tract (20).

In WT C57BL/6 male mice, systemic inhibition of DPP-4 activity with sitagliptin reduced TG excursion following oral olive oil gavage during a lipid tolerance test (LTT) (Figure 1C). Circulating levels of active GLP-1 were increased approximately 4-fold in response to 10 mg/kg sitagliptin (Figure 1C). Plasma levels of active GLP-1 were also induced, albeit to a lesser extent, with lower gut-selective doses of sitagliptin (~2.5-fold vs. 4-fold higher, for gut-selective vs. systemic doses of sitagliptin, respectively) (Figure 1C). Nevertheless, the improvement in lipid tolerance was similar in mice receiving the 2 different doses of sitagliptin (Figure 1C), highlighting the importance of DPP-4^+^ cells within the gut for control of lipid tolerance.

We next used tyloxapol to determine whether the decrease in TG excursion observed with sitagliptin was secondary to a reduction in TRL production or an increase in clearance (21). Plasma TG levels were similarly reduced by the 2 different doses of sitagliptin in the presence of tyloxapol (Figure 1D). Moreover, sitagliptin, at doses that inhibited systemic DPP-4 activity, reduced levels of plasma apoB48, without affecting plasma apoB100 concentrations, consistent with a reduction in intestinal TRL secretion (Figure 1, E and F). In contrast, levels of apoB48 were not reduced by the gut-selective dose of sitagliptin (Figure 1, E and F).

### Genetic elimination of Dpp4 in hematopoietic and endothelial cells increases plasma active GLP-1 and improves lipid tolerance.

DPP-4 is detected in gut blood vessels immediately adjacent to GLP-1^+^ enteroendocrine cells (18). To identify the cellular pool of DPP-4 critical for the reduction of postprandial lipid excursion, we studied (a) mice with whole-body germline inactivation of *Dpp4* (*Dpp4^–/–^*), (b) mice with targeted inactivation of *Dpp4* in Tie2^+^ endothelial and hematopoietic cells (*Dpp4*^EC–/–^), and (c) mice with inactivation of *Dpp4* selectively in Villin^+^ cells (*Dpp4*^Gut–/–^) (20). To differentiate between contributions from endothelial versus hematopoietic cells in *Dpp4*^EC–/–^ mice, we performed bone marrow transplantation (BMT) using WT donor bone marrow transplanted into *Dpp4*^EC–/–^ and *Dpp4*^EC+/+^ mice to produce *Dpp4*^EC–/–^ (BMT) and *Dpp4*^EC+/+^(BMT) mice, respectively (20). *Dpp4*^EC–/–^ (BMT) mice exhibited selective reconstitution of DPP-4 in hematopoietic but not endothelial cells (20).

**Figure 2:**
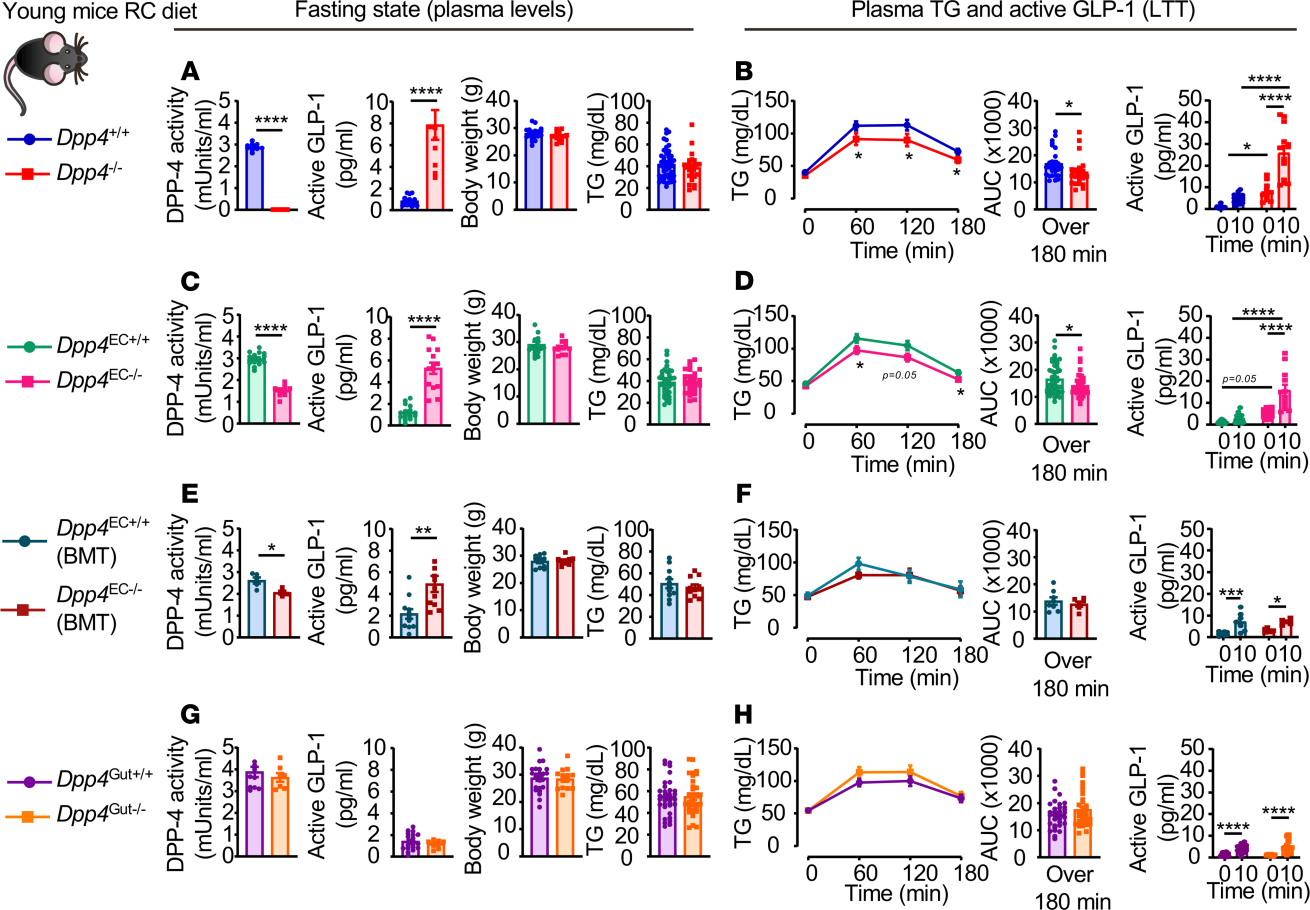
Genetic elimination of *Dpp4* globally or in endothelial and hematopoietic cells increases plasma GLP-1 and improves lipid tolerance in RC-fed mice. Plasma DPP-4 activity, plasma active GLP-1, body weight, and plasma TG after a 5-hour fast (**A**, **C**, **E**, and **G**) and plasma TG and AUC over 180 minutes and plasma levels of active GLP-1 before and 10 minutes after oral gavage of olive oil (**B**, **D**, **F**, and **H**) during a lipid tolerance test (LTT) in 10- to 13-week-old *Dpp4*^–/–^ vs. *Dpp4^+/+^* mice. (**A** and **B**, *n* = 23–30/group for TG, *n* = 10/group for other groups), *Dpp*4^EC–/–^ vs. *Dpp4*^EC+/+^ (**C** and **D**, *n* = 39–47/group for TG, *n* = 10–16 for other groups), *Dpp4*^EC–/–^ (BMT) vs. *Dpp4*^EC+/+^ (BMT) (**E** and **F**, *n* = 6–8/group for TG, *n* = 4–6 for other groups), and *Dpp4*^Gut–/–^ vs. *Dpp4*^Gut+/+^ (**G** and **H**, *n* = 33/group for TG, *n* = 7–11 for other groups) mice fed a regular chow (RC) diet. Data are presented as mean ± SEM. Each *n* represents a biological replicate from 7 (**A** and **B**), 10 (**C** and **D**), and 2 (**E** and **F**) independent cohorts of sex- and age-matched animals. *P < 0.05, **P < 0.01, ***P < 0.001, ****P < 0.0001, using Student’s *t* test (**A**, **C**, **E**, and **G** and left panels in **B**, **D**, **F**, and **H**) or 1-way ANOVA with Tukey’s correction for multiple comparisons (right panels in **B**, **D**, **F**, and **H**), for the indicated groups. BMT, bone marrow transplantation.

RC-fed *Dpp4*^–/–^ mice exhibited no detectable plasma DPP4 activity, an approximately 8-fold increase in plasma active GLP-1, reduced fasting glucose, improved glucose tolerance, and no difference in body weight compared with *Dpp4*^+/+^ mice (Figure 2A and Supplemental Figure 1, A and B; supplemental material available online with this article; https://doi.org/10.1172/jci.insight.140418DS1). Fasting plasma TG levels were similar in *Dpp4*^–/–^ and *Dpp4*^+/+^ mice (Figure 2A); however, postprandial TG excursion was reduced after olive oil administration in *Dpp4*^–/–^ mice (Figure 2B, left). Consistent with these findings, plasma levels of active GLP-1 were increased after olive oil administration in *Dpp4*^–/–^ mice (~4.9 vs. 26.9 pg/mL for *Dpp4*^+/+^ vs. *Dpp4*^–/–^, respectively, Figure 2B, right).

In contrast to findings of improved glycemia in *Dpp4*^–/–^ mice, glucose tolerance was not enhanced in *Dpp4*^EC–/–^ and *Dpp4*^EC–/–^ (BMT) mice, despite reductions in DPP-4 activity of approximately 50% and approximately 25%, respectively (Figure 2, C and E, and Supplemental Figure 1, C–F). Similarly, body weight and plasma TG were not different in *Dpp4*^EC–/–^ mice, despite several-fold increases in fasting plasma GLP-1 levels (Figure 2C). However, TG excursion was reduced in *Dpp4*^EC–/–^ mice, in association with elevated GLP-1 levels, following olive oil administration (Figure 2D).

To elucidate the contributions of hematopoietic versus endothelial cells to the control of TG excursion in *Dpp4*^EC–/–^ mice, we reanalyzed these parameters after BMT. BMT itself increased plasma active GLP-1 levels in control animals (Supplemental Figure 2, B and D). *Dpp4*^EC–/–^ (BMT) mice exhibited an approximately 25% decrease in plasma DPP-4 activity and an approximately 2.5-fold increase in plasma active GLP-1, without differences in body weight or levels of fasting TG, consistent with the importance of DPP-4 expression in endothelial cells (Figure 2E). However, postprandial TG excursion and plasma levels of active GLP-1 were not different between *Dpp4*^EC–/–^ (BMT) and *Dpp4*^EC+/+^ (BMT) mice (Figure 2F). Unexpectedly, these findings reveal a key role for DPP-4 within hematopoietic cells in the control of postprandial lipid excursion.

As gut enterocytes represent a major source of DPP-4 within the gut mucosa, and the key intestinal cell type responsible for lipid uptake and TRL secretion, we studied lipid tolerance in *Dpp4*^Gut–/–^ mice (20). Plasma DPP-4 activity, glucose tolerance, body weight, plasma active GLP-1, and TG levels were not different in RC-fed *Dpp4*^Gut–/–^ mice, whether examined in the fasting state or following olive oil gavage (Figure 2, G and H, and Supplemental Figure 1, G and H).

### Identification of cell types required for DPP4i to control lipid tolerance.

**Figure 3:**
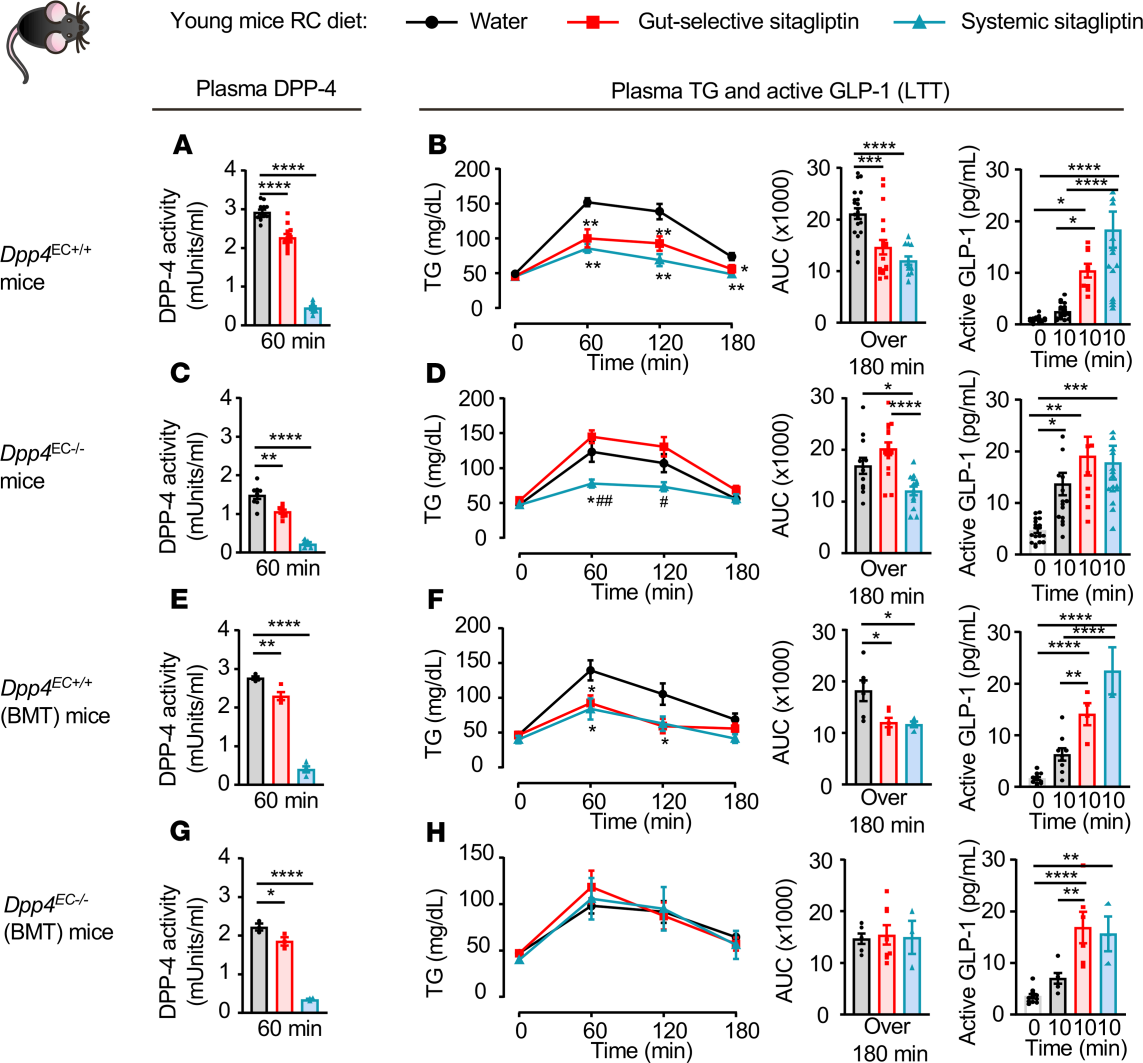
Endothelial and hematopoietic cell–derived DPP-4 is required for the reduction of postprandial lipid excursion by sitagliptin. Plasma DPP-4 activity (**A**, **C**, **E**, and **G**), plasma TG and AUC over 180 minutes (**B**, **D**, **F**, and **H**, left and middle), and plasma levels of active GLP-1 before and 10 minutes after oral gavage of olive oil (**B**, **D**, **F**, and **H**, right) during a lipid tolerance (LTT) in response to water or gut-selective (14 μg/mouse) or systemic (10 mg/kg) dose of sitagliptin in 10- to 13-week-old *Dpp4*^EC+/+^ (**A** and **B**, *n* = 18–22 for TG, *n* = 8–19 for other groups), *Dpp4*^EC–/–^ (**C** and **D**, *n* = 12–16 for TG, *n* = 6–18 for other groups), *Dpp4*^EC+/+^ (BMT) (**E** and **F**, *n* = 6–8 for TG, *n* = 4–12 for other groups), and *Dpp4*^EC–/–^ (BMT) (**G** and **H**, *n* = 6–8 for TG, *n* = 4–12 for other groups) mice fed a regular chow (RC) diet. Data are presented as mean ± SEM. Each *n* represents a biological replicate from 5 (**A**–**D**) and 2 (**E**–**H**) independent cohorts of sex- and age-matched animals. (**A**–**C** and **E**–**H**) *P < 0.05, **P < 0.01, ***P < 0.001, ****P < 0.0001 and (**D**) *P < 0.05 vs. water, ^#^*P* < 0.05 and ^##^*P* < 0.01 vs. gut-selective dose of sitagliptin, using 1-way ANOVA with Tukey’s correction for multiple comparisons for the indicated groups (bar graphs) or compared with the water group (curve graphs, except for **D**). BMT, bone marrow transplantation.

We next assessed the specific DPP-4^+^ cell types essential for transducing the inhibitory actions of DPP4i in control of lipid tolerance. Plasma DPP-4 activity and TG excursion were reduced, and plasma active GLP-1 was increased, in sitagliptin-treated *Dpp4*^+/+^ mice, but sitagliptin had no effect in *Dpp4^–/–^* mice (Figure 1, B and C, and Supplemental Figure 3, A and B), consistent with the selectivity of sitagliptin for DPP-4 (22). A gut-selective dose of sitagliptin reduced lipid excursion and increased active GLP-1 levels in *Dpp4*^EC+/+^ mice but not in *Dpp4*^EC–/–^ mice, although plasma DPP-4 activity was decreased by approximately 20% in both genotypes (Figure 3, A–D). In contrast, systemic inhibition of DPP-4 activity reduced lipid excursion and increased active GLP-1 levels in both genotypes (Figure 3, A–D).

To resolve the contributions of hematopoietic versus endothelial cells to sitagliptin-responsive phenotypes in *Dpp4*^EC–/–^ mice, we repeated the experiments after BMT. Sitagliptin inhibited plasma DPP-4 activity, improved lipid tolerance, and increased plasma active GLP-1 in *Dpp4*^EC+/+^(BMT) mice (Figure 3, E and F). Interestingly, sitagliptin inhibited plasma DPP-4 activity and increased plasma active GLP-1 but failed to improve lipid tolerance in *Dpp4*^EC–/–^ (BMT) mice (Figure 3, G and H).

**Figure 4:**
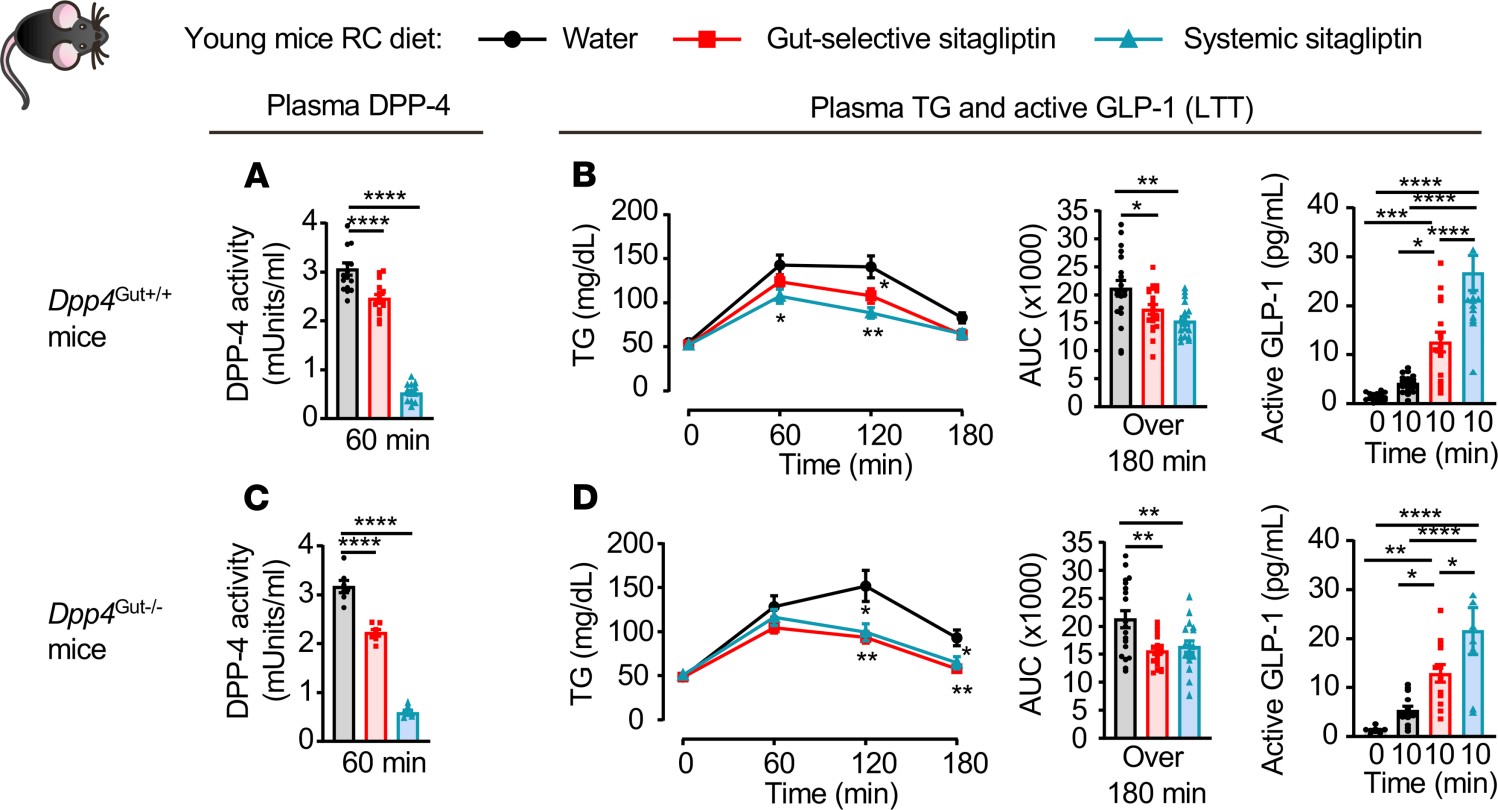
Enterocyte DPP-4 is dispensable for sitagliptin-mediated reduction of postprandial lipid excursion. Plasma DPP-4 activity (**A** and **C**), plasma TG and AUC over 180 minutes (**B** and **D**, left and middle), and plasma levels of active GLP-1 before and 10 minutes after oral gavage of olive oil (**B** and **D**, right) during a lipid tolerance (LTT) in response to water or a gut-selective (14 μg/mouse) or systemic (10 mg/kg) dose of sitagliptin in 10- to 13-week-old *Dpp4*^Gut+/+^ (**A** and **B**, *n* = 14–21/group) and *Dpp4*^Gut–/–^ (**C** and **D**, *n* = 7–19/group) mice fed a regular chow (RC) diet. Data are presented as the mean ± SEM. Each *n* represents a biological replicate from 5 independent cohorts of sex- and age-matched animals. *P < 0.05, **P < 0.01, ***P < 0.001, ****P < 0.0001, using 1-way ANOVA with Tukey’s correction for multiple comparisons for the indicated groups.

We next examined the importance of intestinal DPP-4 as a target for the lipid-regulating activity of sitagliptin by studying *Dpp4*^Gut–/–^ mice. Plasma DPP-4 activity and TG excursion after olive oil administration was reduced, and plasma active GLP-1 was increased to a greater extent after administration of systemically active versus gut-selective doses of sitagliptin in *Dpp4*^Gut+/+^ mice (Figure 4, A and B). Similarly, both gut-selective and systemically active doses of sitagliptin increased plasma levels of active GLP-1 and reduced TG excursion after olive oil gavage in *Dpp4*^Gut–/–^ mice (Figure 4, C and D). Hence, enterocyte DPP-4 is dispensable for the actions of sitagliptin to reduce TG excursion.

### HFD feeding attenuates the lipid-lowering actions of sitagliptin.

**Figure 5:**
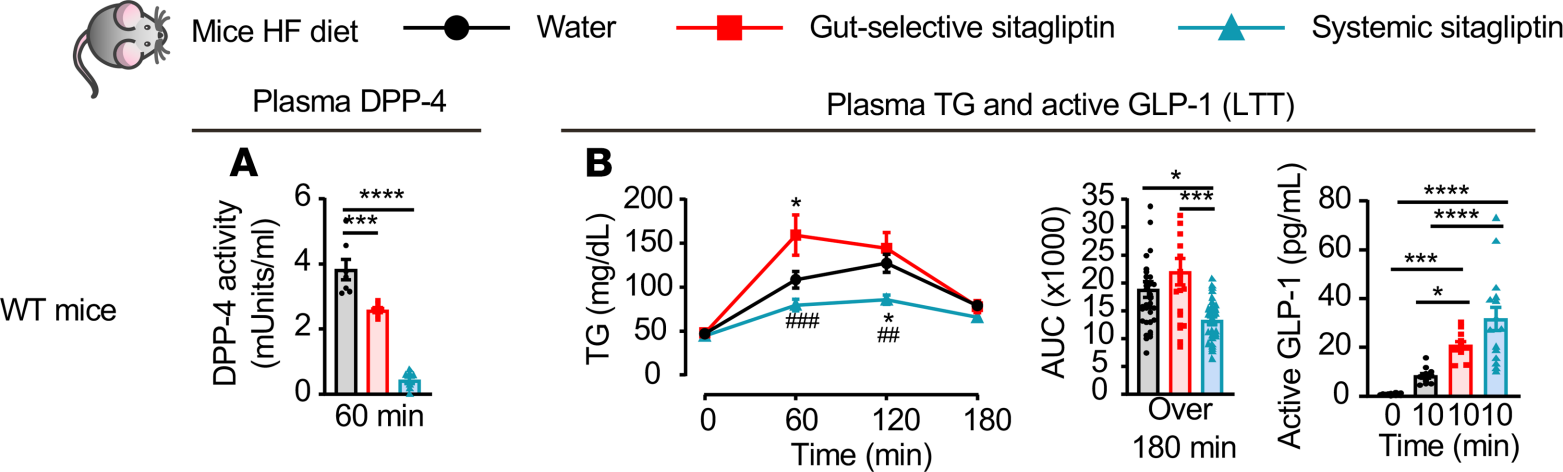
Reduction of DPP-4 activity is not associated with improved lipid tolerance in HFD-fed mice. Plasma DPP-4 activity 60 minutes after olive oil gavage (**A**, *n* = 7/group), plasma TG and AUC over 180 minutes (**B**, left, *n* = 21–35/group), and plasma levels of active GLP-1 30 minutes before (0 minutes) and 10 minutes after olive oil gavage (**B**, right, *n* = 12–15/group) during a lipid tolerance test (LTT) in response to oral gavage of water or a gut-selective (14 μg/mouse) or systemic (10 mg/kg) dose of sitagliptin in 16- to 19-week-old WT mice fed a 45% high-fat (HF) diet for 6–9 weeks. Data are presented as the mean ± SEM. Each *n* represents a biological replicate from 4 independent cohorts of sex- and age-matched animals. (**A**) ***P < 0.001, ****P < 0.0001 and (**B**) *P < 0.05, ***P < 0.001, ****P < 0.0001 vs. water, ^##^*P* < 0.01 and ^###^*P* < 0.001 vs. gut-selective dose of sitagliptin, using 1-way ANOVA with Tukey’s correction for multiple comparisons.

To extend our findings to mice fed an energy-rich HFD, we studied lipid tolerance in C57BL/6 male mice after 6–9 weeks of 45% HFD feeding. In WT mice, systemic inhibition of DPP-4 activity, which inhibited plasma DPP-4 activity by about 90%, improved lipid tolerance and increased plasma active GLP-1 levels by approximately 4.5-fold in HFD-fed mice (Figure 5). Using a gut-selective dose of sitagliptin, plasma DPP-4 activity was reduced by approximately 25% and plasma GLP-1 levels were increased (Figure 5). Nevertheless, despite increased levels of GLP-1, TG excursion was not reduced, and was actually transiently increased, in HFD-fed mice after gut-selective sitagliptin (Figure 5B).

**Figure 6:**
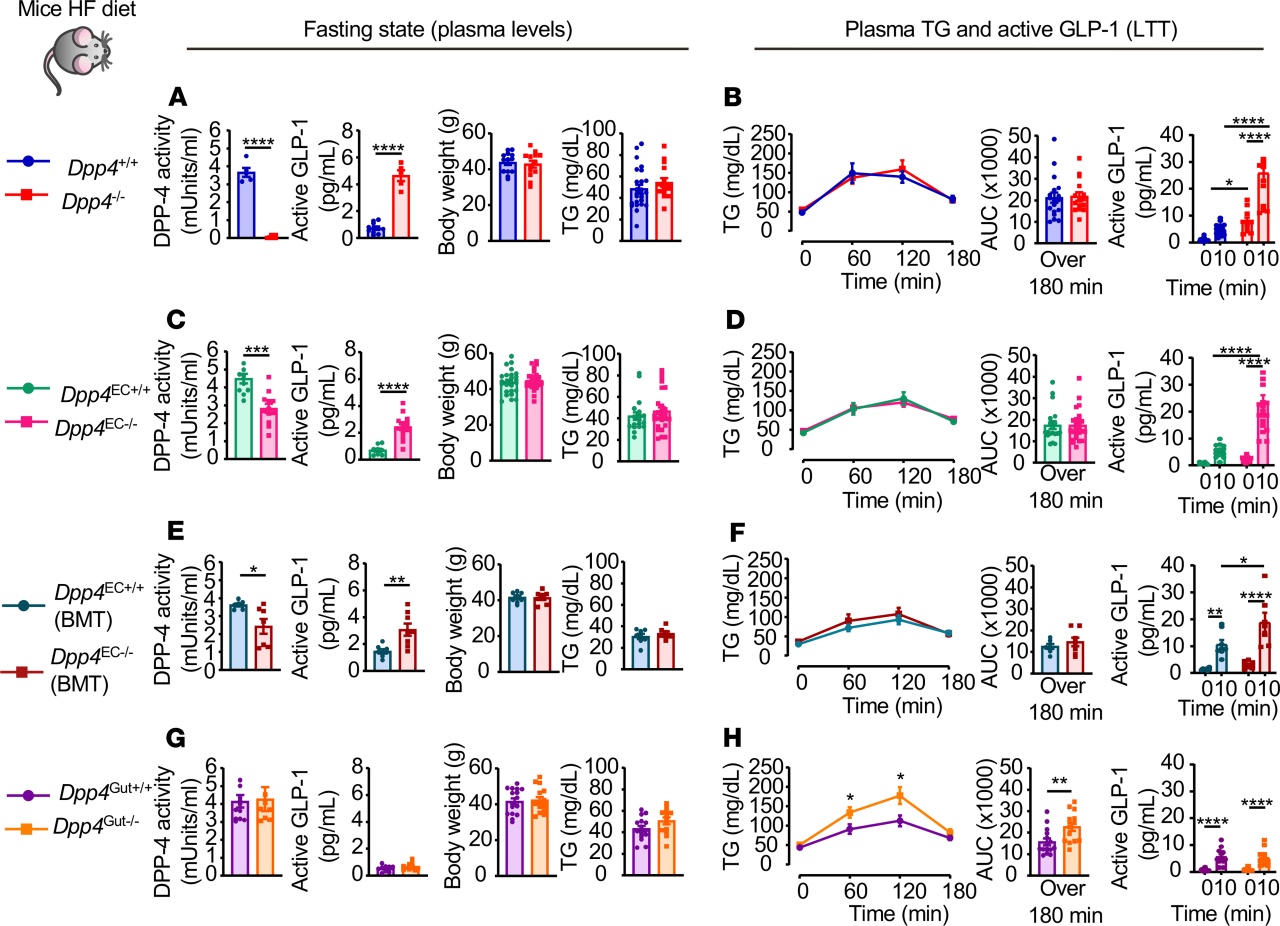
HFD-fed mice exhibit attenuated lipid responses to reduction of DPP-4 activity. Plasma DPP-4 activity, active GLP-1, body weight, and plasma TG after a 5-hour fast (**A**, **C**, **E**, and **G**) and plasma TG, AUC over 180 minutes, and plasma levels of active GLP-1 before and 10 minutes after oral gavage of olive oil (**B**, **D**, **F**, and **H**) during a lipid tolerance test (LTT) in 16- to 19-week-old *Dpp4*^–/–^ vs. *Dpp4^+/+^* (**A** and **B**, *n* = 5–11/group for DPP-4 activity, *n* = 13–18/group for other groups), *Dpp4*^EC–/–^ vs. *Dpp4*^EC+/+^ (**C** and **D**, *n* = 11–12 for DPP-4 activity, *n* = 12–26/group for other groups), *Dpp4*^EC–/–^ (BMT) vs. *Dpp4*^EC+/+^ (BMT) (**E** and **F**, *n* = 7–8/group), and *Dpp4*^Gut–/–^ vs. *Dpp4*^Gut+/+^ (**G** and **H**, *n* = 8–12/group for DPP-4 activity, *n* = 12–16/group for other groups) mice fed a 45% high-fat (HF) diet for 6–9 weeks. Data are presented as the mean ± SEM. Each *n* represents a biological replicate from 6 (**A** and **B**), 5 (**C** and **D**), 1 (**E** and **F**), and 4 (**G** and **H**) independent cohorts of sex- and age-matched animals. *P < 0.05, **P < 0.01, ***P < 0.001, ****P < 0.0001, using Student’s *t* test (**A**, **C**, **E**, and **G** and left panels in **B**, **D**, **F**, and **H**) or 1-way ANOVA with Tukey’s correction for multiple comparisons (right panels in **B**, **D**, **F**, and **H**), for the indicated groups. BMT, bone marrow transplantation.

Furthermore, similar observations were apparent after HFD feeding in other lines of mice. Although DPP-4 activity was reduced, body weight and fasting TG were similar, GLP-1 levels were increased, and lipid tolerance was not improved in HFD-fed *Dpp4*^–/–^, *Dpp4*^EC–/–^, and *Dpp4*^EC–/–^ (BMT) mice (Figure 6, A–F). Surprisingly, plasma TG excursion was actually increased after olive oil administration in HFD-fed *Dpp4*^Gut–/–^ mice, despite a lack of differences in fasting plasma DPP-4 activity, TG levels, and body weight and in fasting or olive oil–induced plasma active GLP-1 levels (Figure 6, G and H).

To probe changes in gut gene expression potentially linked to the lipid phenotypes observed in these mice, we measured mRNA levels in the jejunum corresponding to a subset of genes involved in lipid metabolism. The majority of mRNA transcripts examined were not different in *Dpp4*^–/–^ mice; levels of *Apoe* and *Syb11* were reduced and *Pcsk9* trended higher (Supplemental Figure 4A). Similarly, most mRNA transcripts were similar in *Dpp4*^EC–/–^ versus *Dpp4*^EC+/+^mice, with higher levels of *Apoa4* and *Apob* mRNAs (Supplemental Figure 4B). Gene expression profiles from the proximal jejunum of *Dpp4*^Gut–/–^ mice were largely similar to profiles in *Dpp4*^Gut+/+^ mice; however, *Pcsk9* was higher and levels of *Apoc3* and *Srebf1c* were reduced (Supplemental Figure 4C).

**Figure 7:**
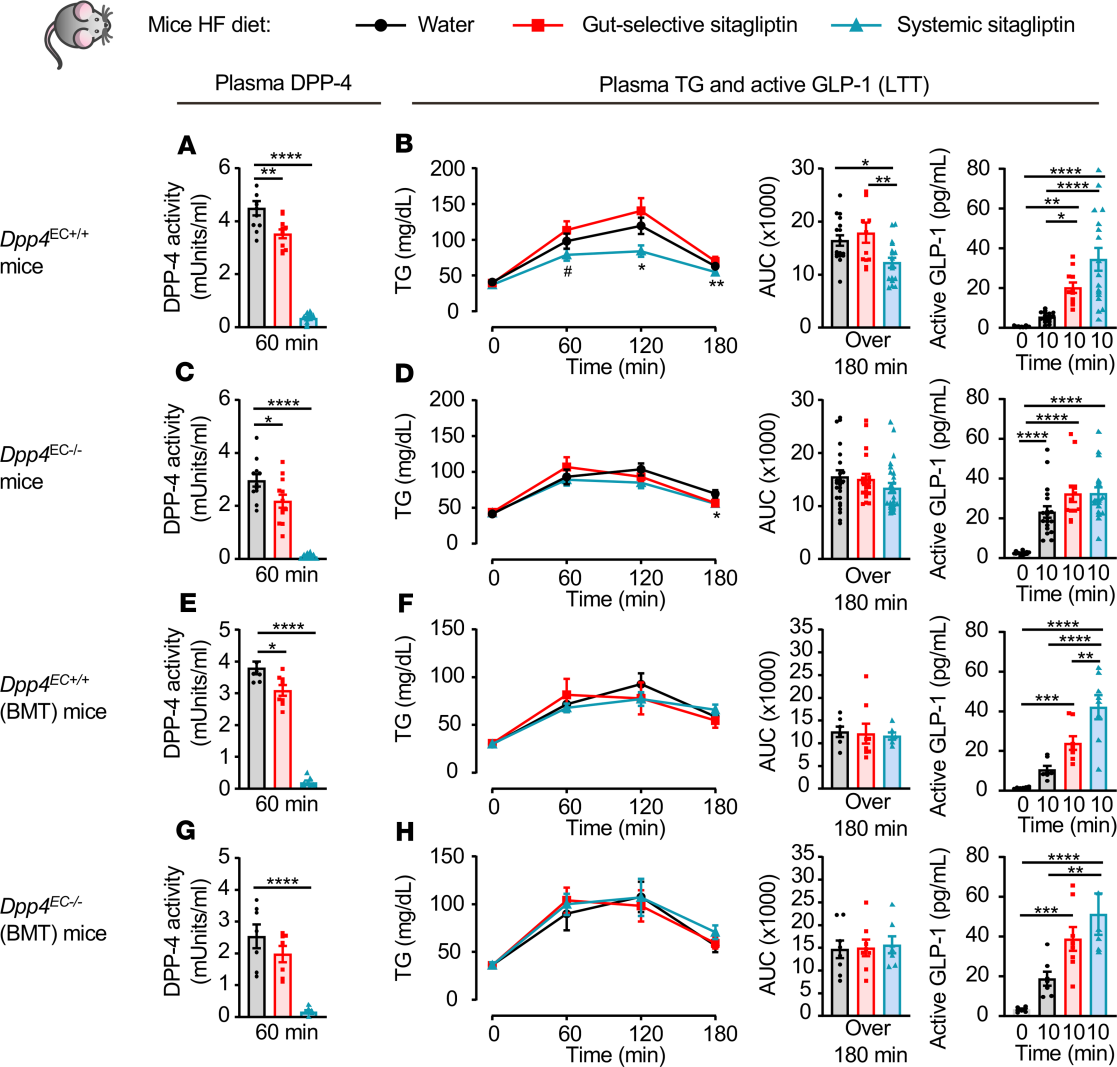
Lipid-lowering actions of sitagliptin are attenuated in HFD-fed *Dpp4*^EC–/–^, *Dpp4*^EC+/+^ (BMT), and *Dpp4*^EC–/–^ (BMT) mice. Plasma DPP-4 activity (**A**, **C**, **E**, and **G**), plasma TG and AUC over 180 minutes (**B**, **D**, **F**, and **H**, left and middle), and plasma levels of active GLP-1 before and 10 minutes after olive oil gavage (**B**, **D**, **F**,and **H**, right) during a lipid tolerance test (LTT) in response to water or a gut-selective (14 μg/mouse) or systemic (10 mg/kg) dose of sitagliptin in 5-hour fasted 16- to 19-week-old *Dpp4*^EC+/+^ (**A** and **B**, *n* = 11–17/group), *Dpp4*^EC–/–^ (**C** and **D**, *n* = 12–26/group), *Dpp4*^EC+/+^ (BMT) (**E** and **F**, *n* = 7–8/group), and *Dpp4*^EC–/–^ (BMT) (**G** and **H**, *n* = 7–8/group) mice fed a 45% high-fat (HF) diet for 6–9 weeks. Data are presented as the mean ± SEM. Each *n* represents a biological replicate from 2–5 independent cohorts of sex- and age-matched animals. *P < 0.05, **P < 0.01, ***P < 0.001, ****P < 0.0001, using 1-way ANOVA with Tukey’s correction for multiple comparisons for the indicated groups. BMT, bone marrow transplantation.

**Figure 8:**
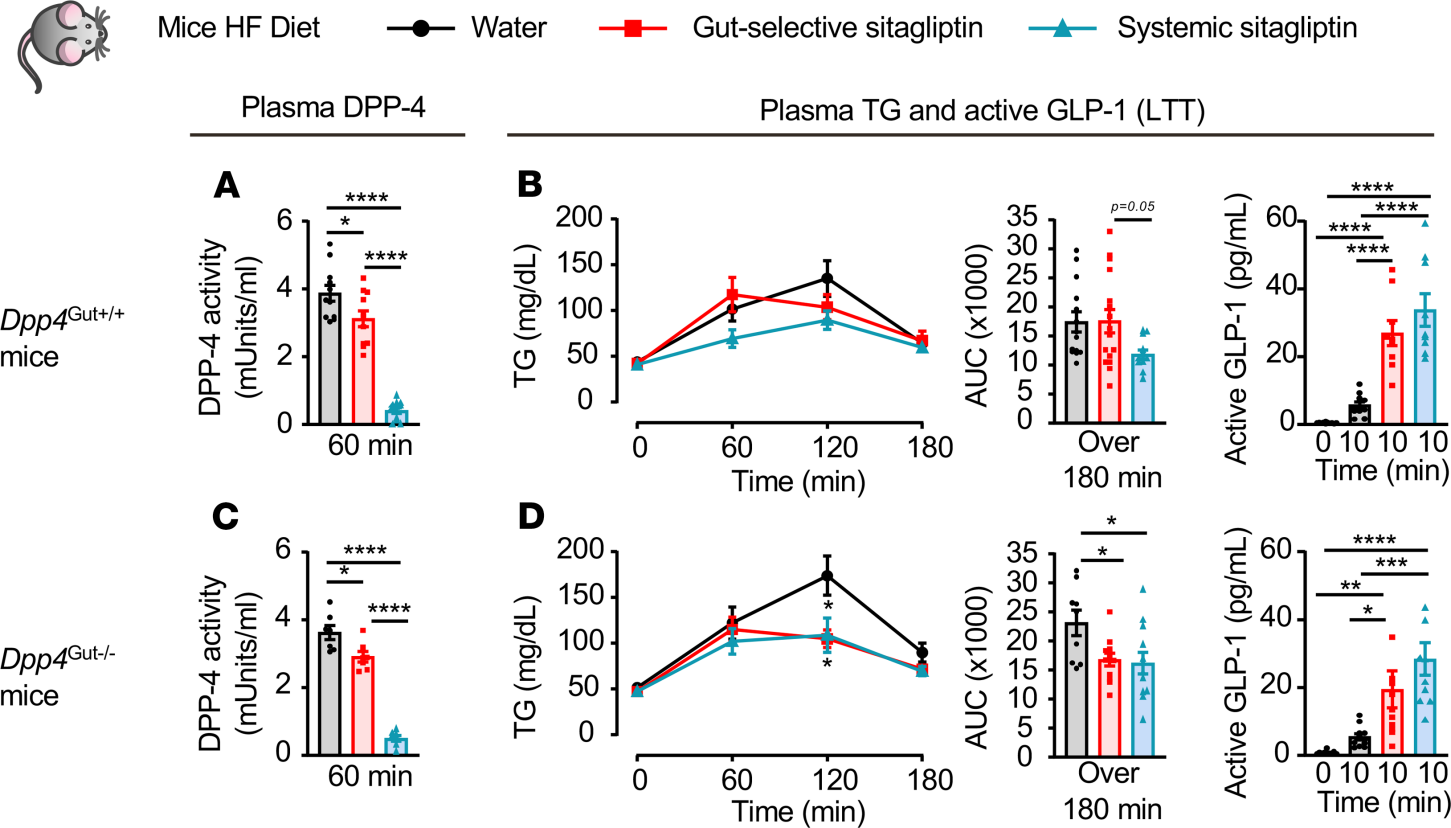
Dysregulation of lipid tolerance in HFD-fed *Dpp4*^Gut–/–^ mice. Plasma DPP-4 activity (**A** and **C**), plasma TG and AUC over 180 minutes (**B** and **D**, left and middle), and plasma levels of active GLP-1 before and 10 minutes after olive oil gavage (**B** and **D**, right) during a lipid tolerance test (LTT) in response to water or a gut-selective (14 μg/mouse) or systemic (10 mg/kg) dose of sitagliptin in 5-hour fasted 16- to 19-week-old *Dpp4*^Gut+/+^ (**A** and **B**, *n* = 9–14/group) and *Dpp4*^Gut–/–^ (**C** and **D**, *n* = 7–11/group) mice fed a 45% high-fat (HF) diet for 6–9 weeks. Data are presented as mean ± SEM. Each *n* represents a biological replicate from 4 independent cohorts of sex- and age-matched animals. *P < 0.05, **P < 0.01, ***P < 0.001, ****P < 0.0001, using 2-way ANOVA with Tukey’s correction for multiple comparisons for the indicated groups.

Consistent with the known specificity of action of selective DPP4i, sitagliptin had no effect on plasma TG excursion or GLP-1 levels in HFD-fed *Dpp4*^–/–^ mice (Supplemental Figure 3, C and D). Moreover, the gut-selective dose of sitagliptin was unable to lower TG excursion in HFD-fed *Dpp4*^EC+/+^ and *Dpp4*^EC–/–^ mice, before and after BMT, despite an approximately 20% reduction in plasma DPP-4 activity and an increase in active GLP-1 (Figure 7). Systemic inhibition of DPP-4 activity reduced lipid excursion and increased active GLP-1 in HFD-fed *Dpp4*^EC+/+^ mice, but not in *Dpp4*^EC–/–^ mice (Figure 7, A–D). In contrast, both gut-selective and systemic inhibition of DPP-4 activity increased GLP-1 and lowered TG excursions in HFD-fed *Dpp4*^Gut–/–^ mice (Figure 8). Hence, intestinal enterocyte-derived DPP-4 is not required for the TG-reducing actions of sitagliptin under RC-or HFD-fed conditions.

### Lipid-lowering effects of sitagliptin are lost in older RC-fed mice.

**Figure 9:**
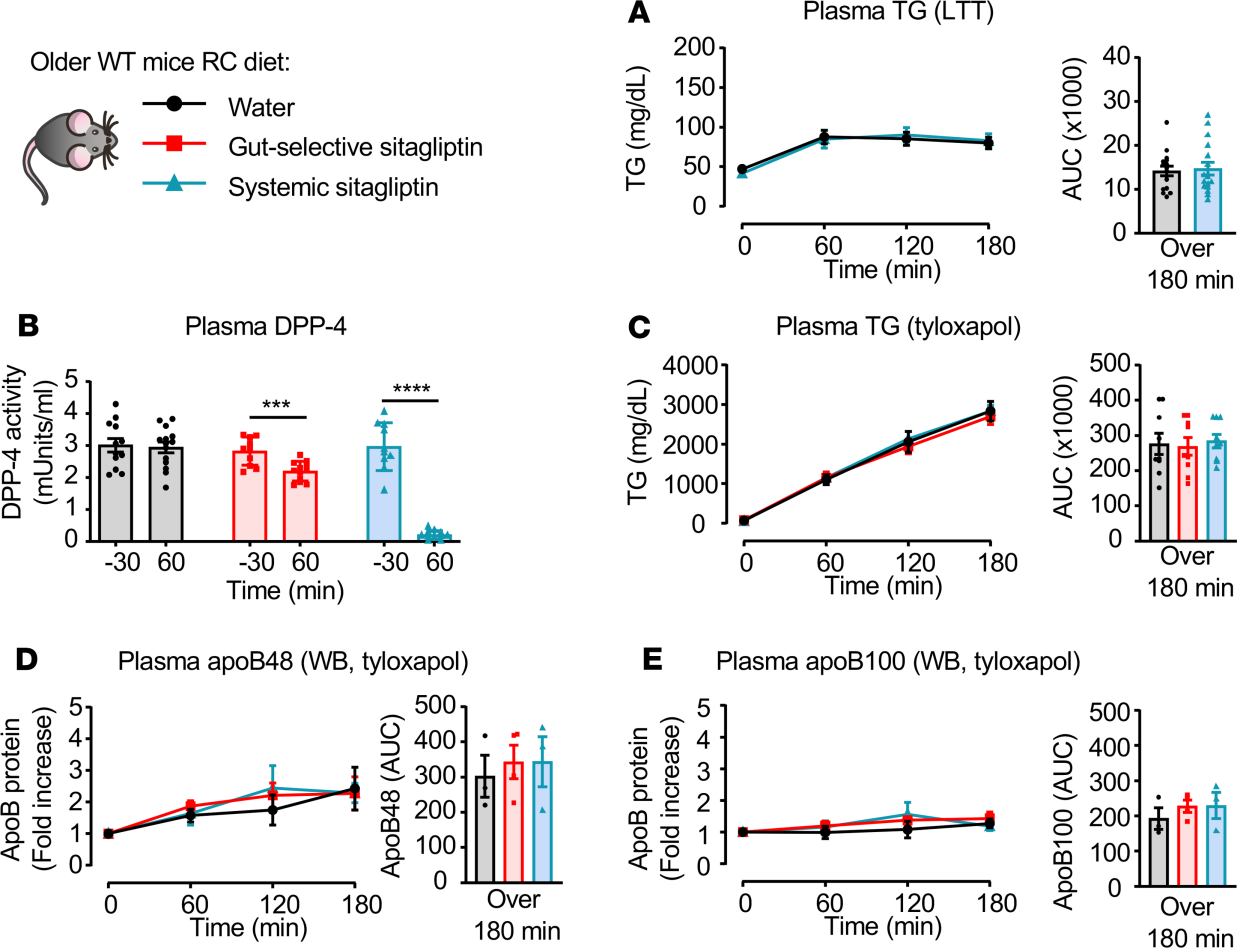
Lipid-lowering effects of sitagliptin are attenuated in older mice. (**A**) Plasma TG and AUC over 180 minutes in 25- to 30-week-old mice fed a regular chow (RC) diet during a lipid tolerance test (LTT) in response to water or a systemic dose of sitagliptin (10 mg/kg) (*n* = 16/group). (**B–E**) Plasma DPP-4 activity before (time –30 minutes) and 90 minutes after oral gavage of water or sitagliptin as indicated (**B**, *n* = 9–14/group), plasma TG and AUC over 3 hours (**C**, *n* = 9–14/group), and apoB48 and apoB100 protein levels measured by Western blot (WB) (**D** and **E**, *n* = 3–5/group) after oral gavage of water or sitagliptin, followed by i.v. injection of 0.5 g/kg tyloxapol and oral gavage of 200 μl olive oil, as described in Figure 1A. Data are presented as mean ± SEM. Each *n* represents a biological replicate from 2 independent cohorts of sex- and age-matched animals. ***P < 0.001, ****P < 0.0001, using Student’s *t* test for the indicated groups.

Given the impairment in DPP4i action evident in HFD-fed mice, we wondered whether DPP-4 inhibition retained its hypolipidemic actions in older mice. Accordingly, we studied 25- to 30-week-old animals fed a RC diet. Surprisingly, despite the reduction in plasma DPP-4 activity, sitagliptin did not lower plasma TG excursions or apoB48 nor apoB100 levels during a LTT, with or without tyloxapol (Figure 9). Similarly, sitagliptin failed to reduce TG production, despite a reduction in plasma DPP-4 activity in older (25–30 weeks of age) *Dpp4*^+/+^, *Dpp4*^–/–^, *Dpp4*^EC+/+^, *Dpp4*^EC–/–^, *Dpp4*^Gut+/+^, and *Dpp4*^Gut–/–^ mice (Supplemental Figure 5, A–D, and Supplemental Figure 6, A and B).

**Figure 10:**
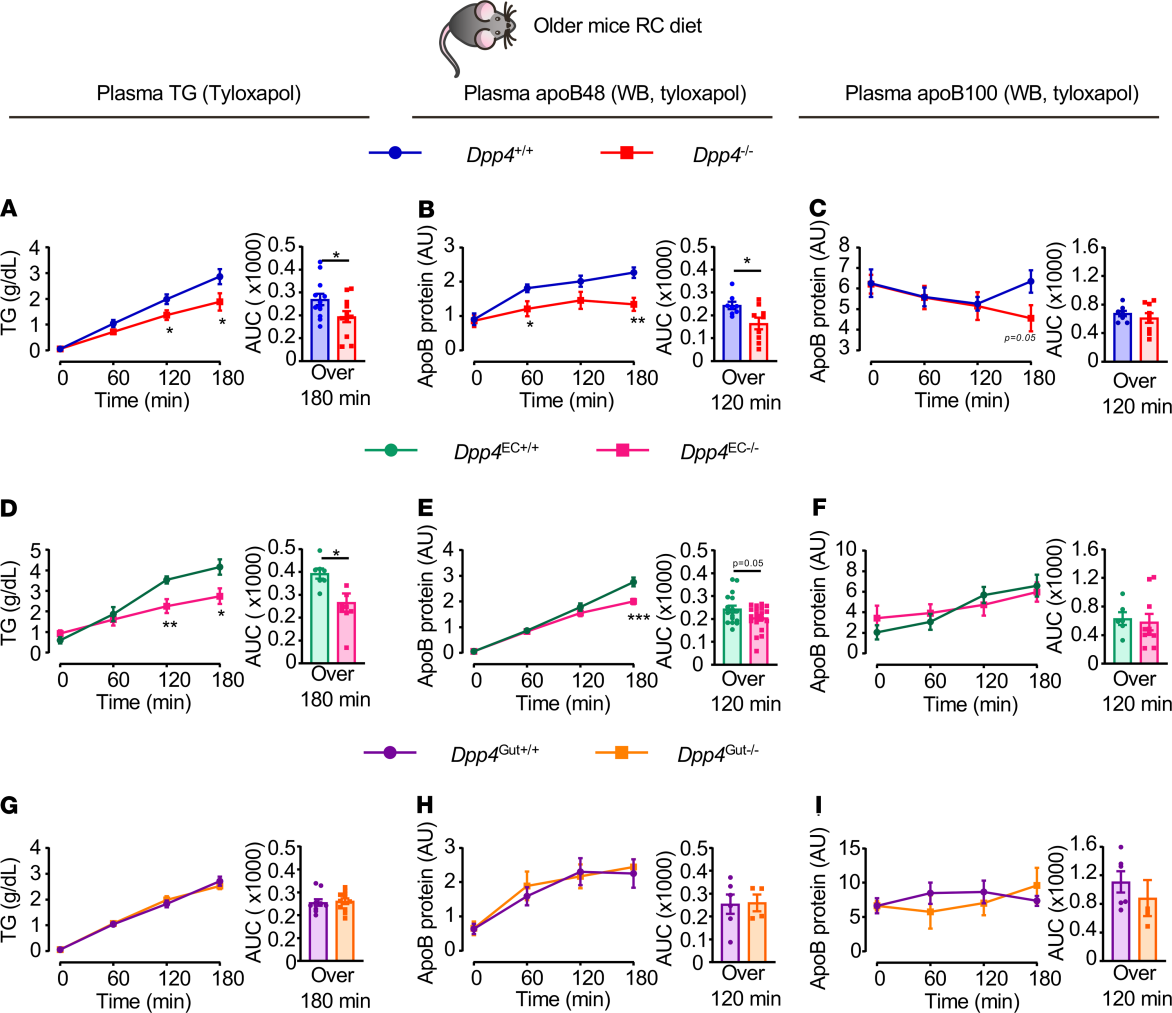
Improved lipid tolerance is preserved in older *Dpp4^–/–^* and *Dpp4*^EC–/–^ mice. Plasma TG and AUC over 180 minutes (**A**, **D**, and **G**) and apoB48 and apoB100 measured by Western blot (WB) (**B**, **C**, **E**, **F**, **H**, and **I**) in response to oral gavage of olive oil (200 μl) and i.v. injection of tyloxapol (0.5 g/kg) in 20- to 25-week-old *Dpp4^–/–^* vs. *Dpp4^+/+^* (**A**–**C**, *n* = 9–12/group), *Dpp4*^EC–/–^ vs. *Dpp4*^EC+/+^ (**D–F**, *n* = 7–20/group), and *Dpp4*^Gut–/–^ vs. *Dpp4*^Gut+/+^ (**G**–**I**, *n* = 4–11/group) mice fed a regular (RC) chow diet. Data are presented as the mean ± SEM. Each *n* represents a biological replicate from 3–4 independent cohorts of sex- and age-matched animals. *P < 0.05, **P < 0.01, ***P < 0.001, using Student’s *t* test for the indicated groups.

In contrast to the diminished response to sitagliptin in older mice, basal TG and apoB48 levels, but not apoB100 levels, remained lower in the presence of tyloxapol in older *Dpp4*^–/–^ and *Dpp4*^EC–/–^ mice after olive oil administration (Figure 10, A–F). Consistent with findings in younger mice, plasma TG, apoB48, and apoB100 were not different in older *Dpp4*^Gut+/+^ versus *Dpp4*^Gut–/–^ mice after olive oil and tyloxapol administration (Figure 10, G–I). Moreover, FPLC lipoprotein fractions were similar in older *Dpp4*^EC–/–^ and *Dpp4*^Gut–/–^ mice and their littermate controls 1 hour after olive oil administration (Supplemental Figure 7, A–D).

## Discussion

The mechanisms and cell types important for DPP-4–mediated control of glucose and islet function have been extensively studied in humans and mice. The available evidence supports the importance of GLP-1 and GIP as key DPP-4 substrates required for potentiating glucose-stimulated insulin secretion and reduction of glycemia following enteral glucose challenge (11–13). Moreover, studies using mouse genetics have identified the importance of endothelial cell DPP-4 in the physiological control of glucose homeostasis and as a key cellular target for the pharmacological glucoregulatory response to DPP-4 inhibition (20). In contrast, DPP-4 expressed within murine hepatocytes, adipocytes, or gut enterocytes is not required for physiological regulation of glucose tolerance and is dispensable for the glucoregulatory actions of DPP4i (20, 23–25).

**Figure 11:**
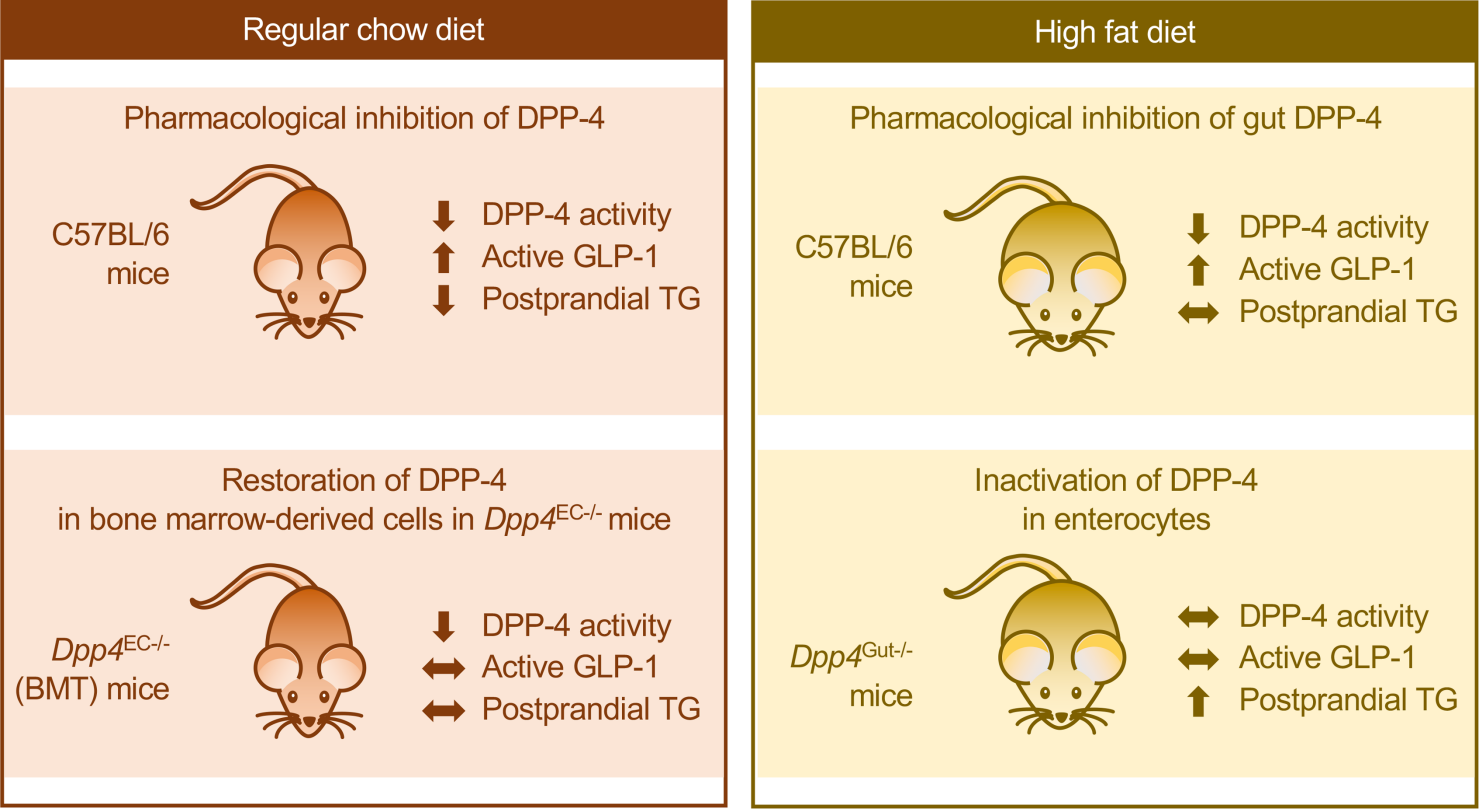
Summary of key messages. In mice studied under regular chow diet conditions, both pharmacological inhibition of local gut DPP-4 and systemic inhibition of DPP-4 activity as well as genetic inactivation of *Dpp4* leads to an increase in plasma active GLP-1 and a reduction in postprandial TG excursion. The relationship among DPP-4 activity, increased GLP-1, and plasma TGs is disrupted in HFD-fed mice. Bone marrow–derived DPP-4 modulates the lipid-lowering effects of DPP4i. Conversely, *Dpp4*^Gut–/–^ mice demonstrate an increase in postprandial TG excursion, notably under high-fat diet conditions. EC, endothelial cells; GLP-1, glucagon like-peptide-1; DPP-4, dipeptidyl peptidase-4; TG, triglycerides.

DPP4i also regulate TG excursion following meals or oral lipid challenge in mice and humans (5, 9, 10, 14). Although insulin is a powerful regulator of lipid metabolism, the acute lipid-lowering actions of GLP-1 have been shown to be mediated independent of changes in islet hormones and glucose and free fatty acid levels in mice and humans (9, 26). However, the key tissues and cell types linking reduction of DPP-4 activity to control of nutrient-stimulated TG excursions have not been previously identified. Here, we show that both gut-selective and systemic inhibition of DPP-4 activity using sitagliptin improves lipid tolerance and systemic doses of sitagliptin robustly reduce intestinal TG production. Unexpectedly, the gut-selective dose of DPP4i failed to reduce apoB48 levels. Our studies also eliminate the possibility that enterocyte-derived DPP-4, accounting for about 90% of gut DPP-4, is the pool responsible for glucose- and lipid-lowering effects of either gut-selective or systemic doses of DPP4i. The results of our studies reveal that bone marrow–derived DPP-4 plays an unexpectedly important role in the lipid-lowering effects of DPP4i (Figure 11).

The pool of circulating DPP-4 in animals fed RC reflects contributions from endothelial and bone marrow cells and osteoclasts but not from enterocytes, hepatocytes, cells from the whole kidney, or adipocytes. In contrast, the increase in circulating DPP-4 induced by HFD originates from hepatocytes (20, 24, 25, 27–30). Nevertheless, the precise cell types contributing DPP-4 to the pool targeted pharmacologically by DPP4i remains incompletely understood and may depend on the experimental model and pharmacodynamic endpoint(s) under investigation.

DPP-4 is highly expressed within enterocytes, the major absorptive epithelial cell type, and genetic elimination of *Dpp4* expression within cells targeted by *Vil1*-Cre reduces intestinal DPP-4 activity by up to approximately 90% (20). Although enterocytes are the major cell type responsible for lipid uptake, chylomicron assembly, and secretion of TRLs (31), loss of enterocyte DPP-4 in *Dpp4*^Gut–/–^ mice does not perturb lipid tolerance in RC-fed mice. Surprisingly, HFD-fed *Dpp4*^Gut–/–^ mice demonstrate increased TG excursion during a LTT, despite olive oil–stimulated increases in circulating levels of GLP-1. These findings raise the possibility that loss of enterocyte DPP-4 in the context of HFD feeding upregulates pathways converging on enhanced TRL secretion. It is possible that loss of GLP-1 responsivity, simultaneous with preservation of GLP-2 activity (32), may shift the enterocyte to a state favoring enhanced lipid secretion, a hypothesis that should be tested in future experiments.

Our molecular analysis of gut mRNA transcript abundance failed to reveal major differences in intestinal mRNA transcripts encoding proteins linked to lipoprotein secretion. Intriguingly, *Pcsk9* mRNA trended higher in *Dpp4^–/–^* mice and was increased in *Dpp4^Gut–/–^* mice, whereas *Pcsk9*^–/–^ mice exhibited reduced postprandial TG, attributed to reduced lymphatic chylomicron secretion and enhanced hepatic clearance (33). Although we detected reduced intestinal levels of *Apoe* mRNA in *Dpp4^–/–^* mice and *Apoc3*, *Dgat2*, and *Srebf1c* expression was decreased in *Dpp4^Gut–/–^* mice, the putative importance of these changes in the context of altered TRL secretion remains unclear.

Most experiments examining the metabolic actions of DPP-4 on glucose and lipid metabolism have been carried out in younger mice. Surprisingly, in contrast to the favorable metabolic activity of sitagliptin evident in younger mice, sitagliptin failed to improve lipid tolerance in older mice. The lipid-lowering actions of sitagliptin were also diminished in HFD-fed mice, despite simultaneous reductions in DPP-4 activity and increased levels of active GLP-1. Collectively, these findings are consistent with the development of relative “GLP-1 resistance” acquired in the context of aging or HFD feeding.

Gut and systemic GLP-1 resistance have been previously described in HFD-fed mice, associated with dysglycemia, insulin resistance, increased food intake, and impaired gastric emptying, and attributed to an acquired impairment of the gut-brain axis secondary to intestinal dysbiosis (34). The CNS control of GLP-1–regulated lipid metabolism in adipocytes was also blunted in HFD-fed mice (35). HFD-induced GLP-1 resistance was associated with reduced intestinal *Glp1r* expression and defective GLP-1–induced activation of murine enteric neuron circuits linked to CNS GLP-1 action (34). Indeed, germ-free mice exhibit reduced intestinal *Glp1r* expression and relative GLP-1 resistance (34) associated with elevated levels of circulating GLP-1 (36), consistent with an important role of the microbiota in determining GLP-1 sensitivity.

Although the concept of GLP-1 resistance in humans has not been extensively explored, substantial interindividual differences in β cell responses to GLP-1 infusion have been described in healthy humans (37), findings correlated with the degree of concomitant insulin resistance. Lack of responsiveness to GLP-1 has also been described in individuals with defective β cell function, including subjects harboring genetic variants of *TCF7L2* (38). Indeed, attenuated clinical responses to DPP4i in humans with T2D are associated with the extent of insulin resistance (39). Renal GLP-1 resistance, associated with attenuated natriuretic responses to GLP-1 has been observed in subjects with T2D (40, 41). Intriguingly, studies in rats have correlated the diminution of GLP-1–stimulated natriuresis in hypertensive animals with a reduction of GLP-1R expression in the kidney (42, 43).

Our current findings in aging or HFD-fed mice with abrogation of improved lipid tolerance, despite reduced DPP-4 activity and increased GLP-1 levels, suggest that the concept of GLP-1 resistance should be further explored in studies examining the GLP-1–dependent control of TG excursion. DPP-4 not only cleaves and inactivates GLP-1, but also regulates the biological activities of GLP-2, a peptide known to enhance intestinal lipid absorption and/or chylomicron secretion (9, 44–46). Furthermore, the incretin hormone GIP enhances lipid uptake into adipose tissue (47), an action which appears impaired in subjects with obesity (48). Moreover, GIP levels are increased in older people (49), and in subjects with T2D and obesity, and correlate with levels of fasting and postprandial TGs (50, 51). Hence, it remains possible that the lipid-lowering actions of GLP-1 are opposed by increased activity of 1 or more DPP-4 substrates that retain their activity in older mice or after HFD feeding.

An unexpected finding in our studies was the loss of improved lipid tolerance in *Dpp4*^EC–/–^ mice, with or without coadministration of sitagliptin, following BMT, implying an interaction between bone marrow–derived cells and the metabolic actions emanating from the reduction of DPP-4 activity in endothelial cells. As sitagliptin clearly reduced DPP-4 activity and increased GLP-1 levels in mice after BMT, these findings are consistent with impairment of GLP-1 action by bone marrow–derived cells. Recent studies have identified a role for bone marrow precursors in the trafficking of immune cells to the gut, enabling development of intestinal intraepithelial lymphocytes (IELs) within the gut that express the GLP-1R (52). Genetic disruption of lymphocyte trafficking induces a local form of GLP-1 resistance, with depletion of GLP-1R^+^ IELs accompanied by a marked rise in intestinal GLP-1 production. Consistent with these studies, our current findings, showing a notable increase in plasma active GLP-1 levels after BMT in control and *Dpp4*^EC–/–^ mice, imply that BMT may somehow confer GLP-1 resistance in *Dpp4*^EC–/–^ mice. Understanding which bone marrow cells are reconstituted and responsive to a DPP4i and how these cells may influence GLP-1 action and TG metabolism in the gut represents an important focus for further studies.

Our current findings have several limitations. First, we studied several lines of mice with germline inactivation of the *Dpp4* gene, invoking the possibility of compensation that might modify the observed phenotypes. Second, the majority of our studies were acute in nature and carried out in mice without chronic hyperglycemia, limiting the extrapolation of our data to settings using chronic administration of DPP4i in diabetic mice. Third, we did not incorporate systemic analyses of hepatic lipid metabolism in the elucidation of TG excursion, thus excluding potential hepatic contributions to the interpretation of our data. Moreover, we did not prospectively collect stool samples for analysis of possible changes in the gut microbiome linked to mouse phenotypes. Taken together, our current data advance the understanding of the cellular sites of DPP-4 action linked to the control of TG excursion. These data also raise the possibility of tissue GLP-1 resistance as a key factor underlying the failure to improve lipid tolerance in mouse models characterized by reduced DPP-4 activity, elevated levels of GLP-1, and yet normal or deteriorated lipid tolerance.

## Methods

### Animals.

C57BL/6 male mice were housed (2–5 mice per cage) under a 12-hour-light/dark cycle in the Toronto Centre for Phenogenomics (TCP) facility. WT control mice originated from an in-house mouse colony. *Dpp4^–/–^* mice were rederived from a colony described previously (53). *Dpp4*^fl/fl^ mice were obtained from Merck Laboratories; the LoxP sites of this mouse encompass the catalytic serine in exon 22. B6.Cg-Tg(Tek-cre)1Ywa/J (*Tie2*-Cre), B6.SJL-Tg(*Vil1*-Cre)997Gum/J (*Vil*-Cre), and B6.SJL-Ptprc^a^ Pepc^b^/BoyJ mice (for bone marrow transplant) were obtained from The Jackson Laboratory. As described for the B6.Cg-Tg(Tek-cre)1Ywa/J strain (54), germline deletion was prevented by restricting *Cre* expression to male breeders. Conversely, as B6.SJL-Tg(*Vil1*-cre)997Gum/J mice demonstrated germline deletion when *Cre* was expressed in male breeders, female breeders were used for *Cre* expression in this line. To control for gene dosage, breeders were heterozygous for the *Cre* gene. Intercrossing *Cre*^+^ and *Cre*^–^ *Dpp4* loxP heterozygotes resulted in 4 genotypes: WT mice with no *Cre* recombinase, mice homozygous for LoxP sites within the *Dpp4* gene (*Dpp4*^fl/fl^), WT mice expressing *Cre* recombinase (*Tie2-Cre* and *Vil1-Cre*), and *Dpp4*^fl/fl^ mice expressing *Cre* recombinase (*Dpp4*^EC–/–^ and *Dpp4*^Gut–/–^). All mice were born at the expected Mendelian ratios and appeared healthy. All mice were maintained on RC (8% kcal from fat; 2018, Harlan Teklad) or HFD (45% kcal fat, 35% kcal carbohydrate, 0.05% wt/wt cholesterol, D12451, Research Diets) with free access to food and water unless otherwise noted.

Whenever possible, we carried out experiments in all groups of mice and age-matched littermate controls. Due to the lack of phenotypic differences in metabolic parameters within the control lines (20), we present data for control mice (aggregate of mice expressing Cre (*Vil1*-Cre for *Dpp4*^Gut–/–^ and *Tie2*-Cre for *Dpp4*^EC–/–^) and *Dpp4*^fl/fl^ mice (littermate controls from the corresponding line) versus mice with the 2 different targeted *Dpp4* deletions. Mice were fasted for 5 hours before metabolic studies and sacrifice.

### LTTs and TG production measurement.

After a 5-hour fast, mice were given water or sitagliptin (Merck Laboratories) by oral gavage at 14 μg/mouse (for intestinal-selective inhibition of DPP-4, ref. 20) or 10 mg/kg (for systemic inhibition of DPP-4). Thirty minutes later, mice were gavaged with 200 μL olive oil (MilliporeSigma). For TG production measurement only, mice were fasted for 4 hours (bled the day before for baseline *t*0 values to avoid bleeding during the i.v. injection) and administered tyloxapol (0.5 g/kg body weight from a 15% solution [3.3 times body weight]) by i.v. injection immediately before olive oil gavage. Blood was collected from the tail vein up to 3 hours after gavage to measure TG levels and other metabolic parameters (40–75 μl per time point). As TGs accumulate in the circulation after tyloxapol injection, these experiments were terminal, and mice were sacrificed after the 3-hour time point. For LTT experiments, each mouse received all 3 treatments (water, gut-selective DPP4 dose, or systemic DPP4 dose), separated from each other by 6–10 days. For experiments using tyloxapol, mice were randomized to treatment group based on genotype, sex, age, and body weight before the experiment.

### Analysis of lipoprotein particles by FPLC.

Mice (18–24 weeks old) were fasted for 5 hours. Two hundred μl of olive oil was given orally at time 0, and approximately 100 μl of blood was collected from the tail vein into EDTA-coated capillary tubes 60 minutes later. Blood was centrifuged at 18,000*g* for 5 minutes at 4°C, and plasma was collected. Plasma lipoproteins were separated by FPLC, and collected fractions were assayed for TG and cholesterol as previously described (55).

### Oral glucose tolerance tests.

After a 5-hour fast, mice were administered water or sitagliptin at 14 μg/mouse (gut-selective dose) or 10 mg/kg (systemic DPP-4 inhibition) by gavage. Thirty minutes later, RC-fed mice were gavaged with 20% glucose in PBS (2 g/kg of body weight) and HFD-fed mice were gavaged with 50% glucose in PBS (2 g/kg of body weight). Blood samples for glucose measurements (Contour glucometer, Bayer Healthcare) were taken from the tail vein up to 120 minutes after injection.

### Blood and tissue collection and metabolic assays.

For metabolic studies, blood was taken via the tail vein into heparin-coated capillary tubes. For DPP-4 activity assays, blood was collected before and 30 minutes or 1 hour after olive oil gavage, and activity was assessed using a fluorometric assay (substrate: 10 mM H-Gly-Pro-AMC HBr [Bachem, I-1225], standard: AMC [Bachem, Q-1025]). For the measurement of active GLP-1 (Mesoscale, 150JVC-1), blood was taken before treatment (*t* –30 minutes) and 10 minutes after olive oil gavage and mixed with 10% TED (5000 KIU/mL Trasylol, 1.2/mL mg/mL EDTA, and 0.1 nmol/L Diprotin A). For TG measurements, blood was taken before and 1, 2, and 3 hours after olive oil gavage. Plasma was isolated and stored at –80°C until analysis.

At the end of the study, mice were sacrificed by CO_2_ inhalation, blood was obtained by cardiac puncture, and plasma was stored at –80°C. Tissues for analysis were snap frozen in liquid nitrogen and stored at –80°C.

### BMT.

Bone marrow chimeras were generated by lethally irradiating *Dpp4*^EC+/+^ (pool of *Dpp4*^fl/fl^ and *Tie2*-Cre mice) and *Dpp4*^EC–/–^ mice (1100 cGy split into 2 equal doses 4 hours apart), followed by reconstitution with 5 × 10^6^ bone marrow cells from donor B6.SJL-Ptprc^a^ Pepc^b^/BoyJ mice as described previously (56). After 8 weeks, the efficiency of reconstitution was assessed by flow cytometry analysis of blood using CD45.1 or CD45.2 antibodies as described previously (20).

### Plasma apoB quantification.

ApoB48 and apoB100 immunoblotting was performed on diluted plasma (1:100) by SDS-PAGE analysis, as previously described (9), using goat anti-apoB antibody (Midland Bioproducts, MBC-APB-G1 [also 71301]; RRID:AB_2734118) and mouse anti-goat IgG-HRP (Santa Cruz Biotechnologies, sc-2354; RRID:AB_628490). Membranes were incubated in electrochemiluminescence detection reagents (Super Signal West Pico PLUS Chemiluminescent Substrate, Thermo Scientific, P134577) and read using a ChemiDoc XRS^+^ (Bio-Rad). Quantitative analysis was performed using ImageJ software (NIH).

### RNA analyses.

First-strand cDNA was synthesized from total RNA using the SuperScript III synthesis system (Invitrogen). Proximal jejunum mRNA abundance was determined using a 2-step quantitative real-time PCR (qRT-PCR) protocol on an ABI Prism Sequence Detection System (Applied Biosystems, model 7900 HT) according to the manufacturer’s instructions. Primer probe sets were from TaqMan Assays-on-Demand (Applied Biosystems) and are detailed in Supplemental Table 1. The standard curve method was used to determine mRNA concentrations, and each gene was normalized to cyclophilin (*Ppia*) expression.

### Statistics.

Results are expressed as the mean ± SEM. Statistical comparisons were made by 1- or 2-way ANOVA followed by Tukey’s post hoc or by 2-tailed unpaired Student’s *t* test (only 2 conditions) using GraphPad Prism 8. A *P* value of less than 0.05 was considered significant. Significant outlier data points were detected using the Grubbs’ test and excluded from the analysis.

### Study approval.

All experiments were approved (animal use protocol approval 20-0045H) by the animal care and use subcommittee at the TCP at Mt. Sinai Hospital.

## Author contributions

Research study design was planned by EMV, EEM, and DJD. EMV, EEM, AAH, MAN, JLB, XC, and LLB conducted the experiments. EMV and EEM acquired, analyzed, and graphed the data. The original draft was written by EMV, EEM, and DJD and was reviewed and edited by EMV, EEM, JLB, LLB, and DJD. DJD supervised this project.

## Supplementary Material

Supplemental data

## Version 1 07/14/2020

In-Press Preview

## Version 2 08/20/2020

Electronic publication
