# Supplementary material for: Hematopoietic cell– versus enterocyte-derived dipeptidyl peptidase-4 differentially regulates triglyceride excursion in mice
Source: JCI Insight. 2020 Aug 20;5(16):e140418. doi: 10.1172/jci.insight.140418 (PMC7455127; doi:10.1172/jci.insight.140418)
Supplement: Supplemental data [file jciinsight-5-140418-s253.pdf]

Supplemental Figure 1

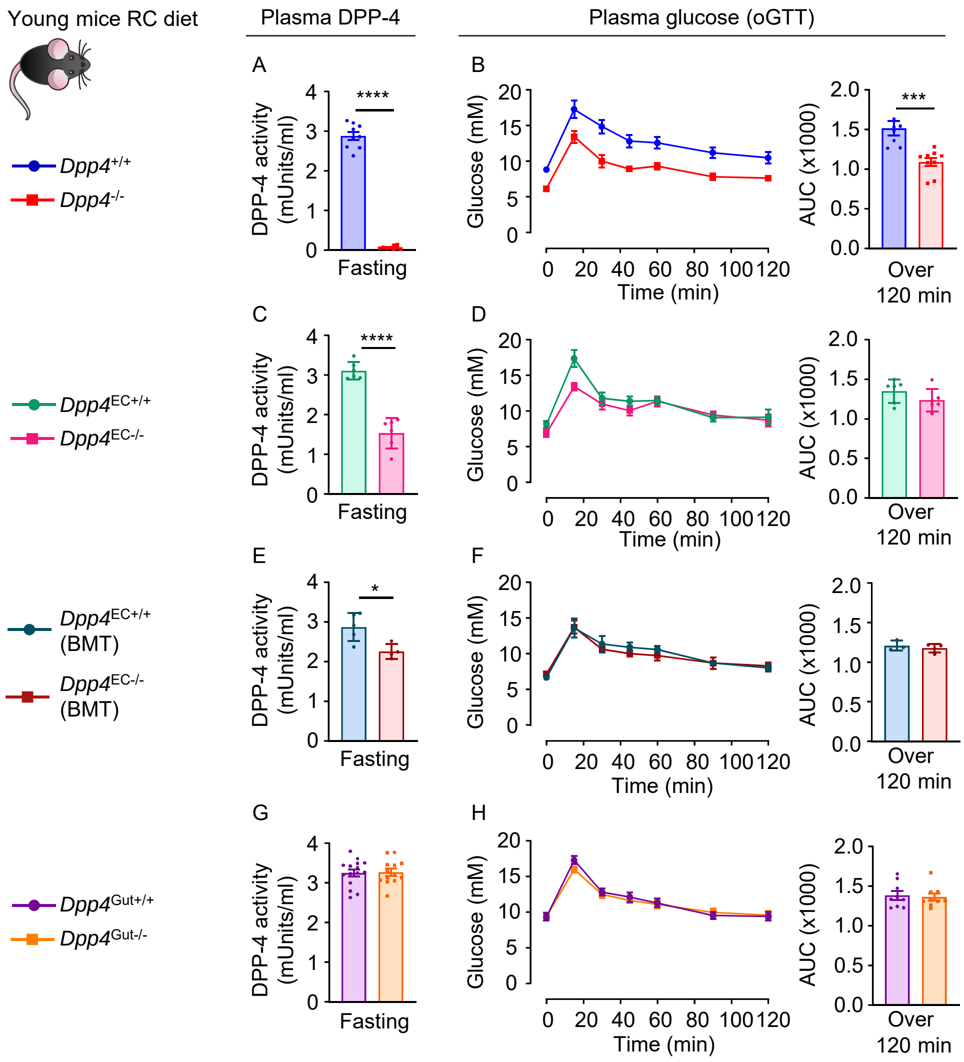

Supplemental Figure 1. Glucose tolerance is improved in whole body *Dpp4*$^{-/-}$ RC-fed mice. A-H: Fasting plasma DPP-4 activity (A,C,E,G), and glucose levels and AUC over 120 minutes (B,D,F,H) after oral gavage of 2g/kg glucose during an oral glucose tolerance test (oGTT) in 10-14 week-old *Dpp4*$^{-/-}$ vs *Dpp4*$^{+/+}$ (A,B, n=5-6/group), *Dpp4*$^{EC-/-}$ vs *Dpp4*$^{EC+/+}$ (C,D, n=8-9/group), *Dpp4*$^{EC-/-}$ (BMT) vs *Dpp4*$^{EC+/+}$ $^{(BMT)}$ (E,F, n=3-4/group), and *Dpp4*$^{Gut-/-}$ vs *Dpp4*$^{Gut+/+}$ (G,H, n=5-6/group) mice fed regular chow (RC) diet. Data are presented as the means ± SEM. Each n represents a biological replicate from 1 cohort each of sex- and age-matched animals. *p<0.05, ***p<0.001, ****p<0.0001 using Student's t-test.

Supplemental Figure 2

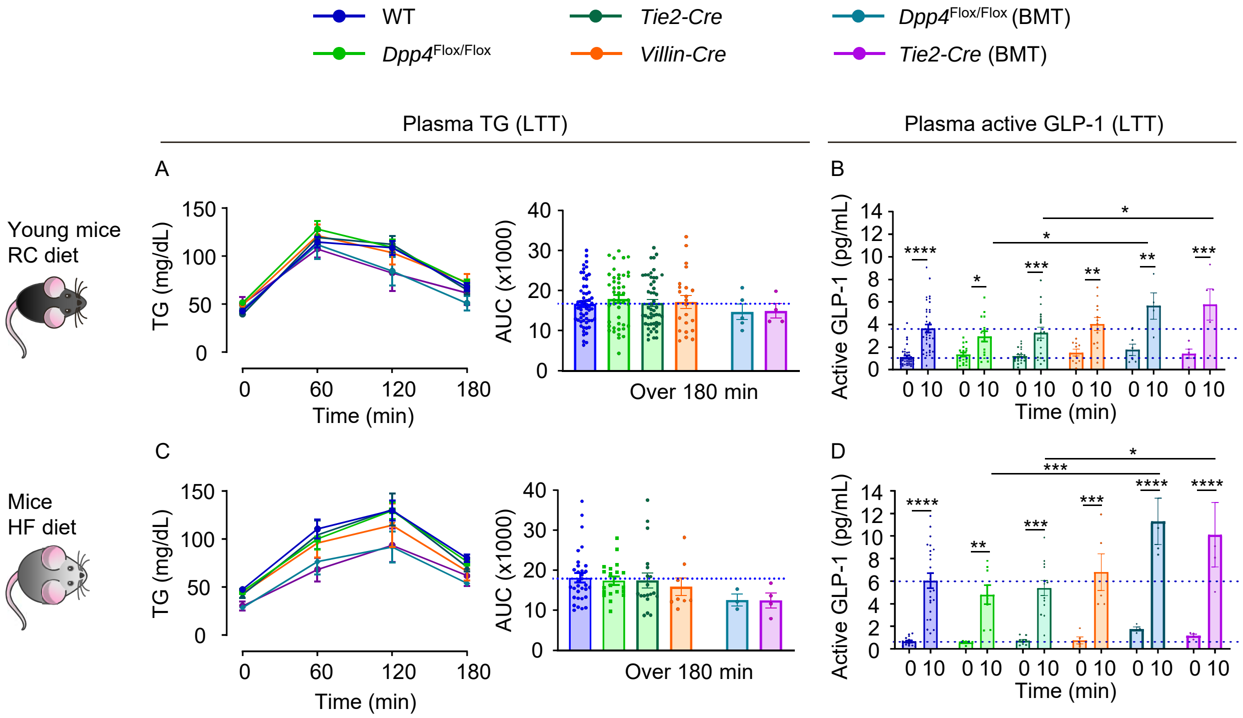

Supplemental Figure 2. Lipid tolerance and active GLP-1 in control mice. A-D: Plasma TG and AUC over 180 minutes (A,C), and plasma active GLP-1 30 minutes before (0) and 10 minutes after oral gavage of olive oil (B,D) during a lipid tolerance test (LTT) in WT (n=32-55 for LTT, n=25-19 for GLP-1), *Dpp4*$^{Flox/Flox}$ (n=20-45 for LTT, n=8-20 for GLP-1), *Tie2*-Cre (n=19-50 for LTT, n=13-24 for GLP-1), *Villi*n-Cre (n=8-22 for LTT, n=18-19 for GLP-1), *Dpp4*$^{Flox/Flox}$ (BMT) (n=3-5) and *Tie2*-Cre (BMT) (n=4) mice fed a regular chow (RC) diet (A,B) or 45% high fat (HF) diet (C,D). Data are presented as the means ± SEM. Each n represents a biological replicate from 10 independent cohorts of sex- and age-matched animals. *p<0.05, **p<0.01, ***p<0.001 ****p<0.0001, two-way ANOVA with Tukey or Sidak correction for multiple comparisons for each indicated groups.

Supplemental Figure 3

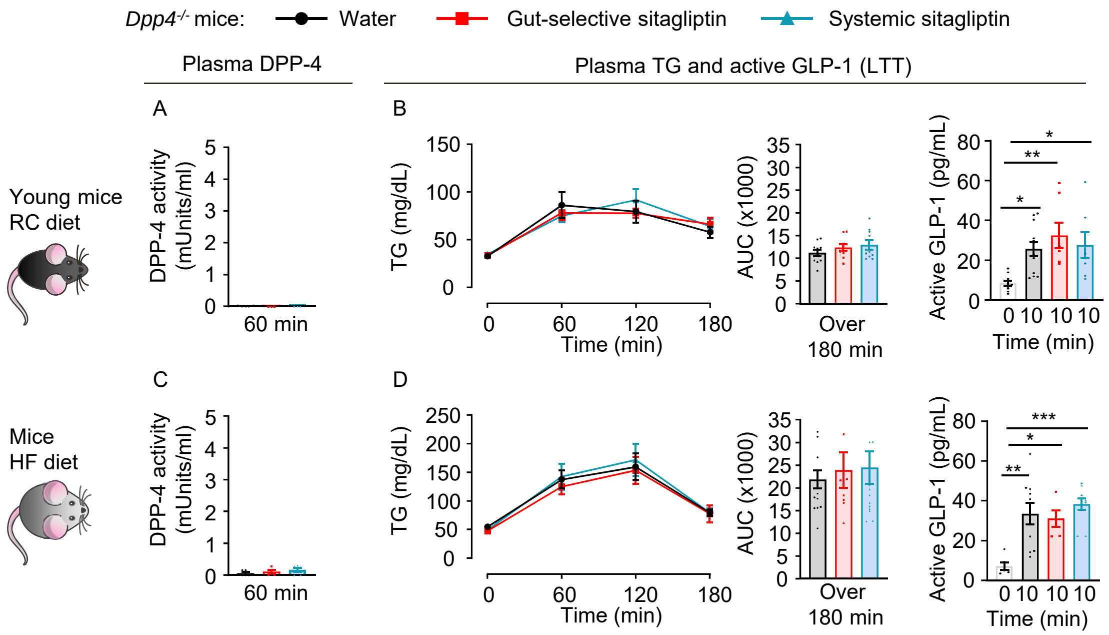

Supplemental Figure 3. Sitagliptin administration and lipid tolerance in *Dpp4$^{-/-}$* mice. A-D: Fasting plasma DPP-4 activity 60 minutes after oil gavage (A,C), plasma TG levels and AUC over 180 minutes (B,D left panels) and plasma active GLP-1 30 minutes before (0) and 10 minutes after oil gavage (B,D right panels), during an oral lipid tolerance test (LTT) in 10-14 week-old *Dpp4$^{-/-}$* mice fed regular chow (RC) diet (A,B, n=7-12/group) or in 16-19 week-old *Dpp4$^{-/-}$* (C,D, n=5-15/group) mice fed high fat (HF) diet for 6-9 weeks. Data are presented as the means ± SEM. Each n represents a biological replicate from 2-5 independent cohorts of sex- and age-matched animals. *p<0.05, **p<0.01, ***p<0.001 using one-way ANOVA with Tukey correction for multiple comparisons for each indicated groups.

Supplemental Figure 4

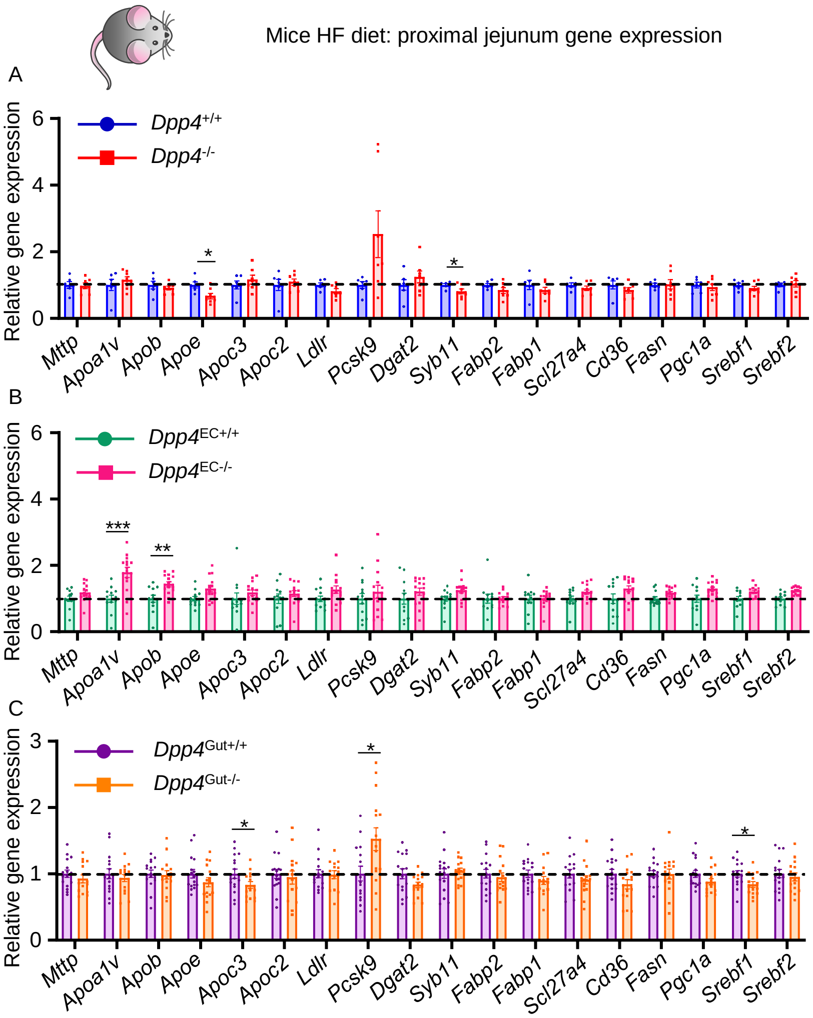

Supplemental Figure 4. Intestinal gene expression related to lipid metabolism. A-C: mRNA expression profile of genes involved in lipid metabolism in 16-19 week-old *Dpp4*$^{-/-}$ vs *Dpp4*$^{+/+}$ (A, n=6-7/group), *Dpp4*$^{EC-/-}$ vs *Dpp4*$^{EC+/+}$ (B, n=12-14/group), and *Dpp4*$^{Gut-/-}$ vs *Dpp4*$^{Gut+/+}$ (C, n=15-16/group) mice fed high-fat (HF) diet for 20 weeks. Data are presented as the means ± SEM. Each n represents a biological replicate from 4-6 independent cohorts of sex- and age-matched animals. *p<0.05, **p<0.01, ***p<0.001, using Student's t-test.

Supplemental Figure 5

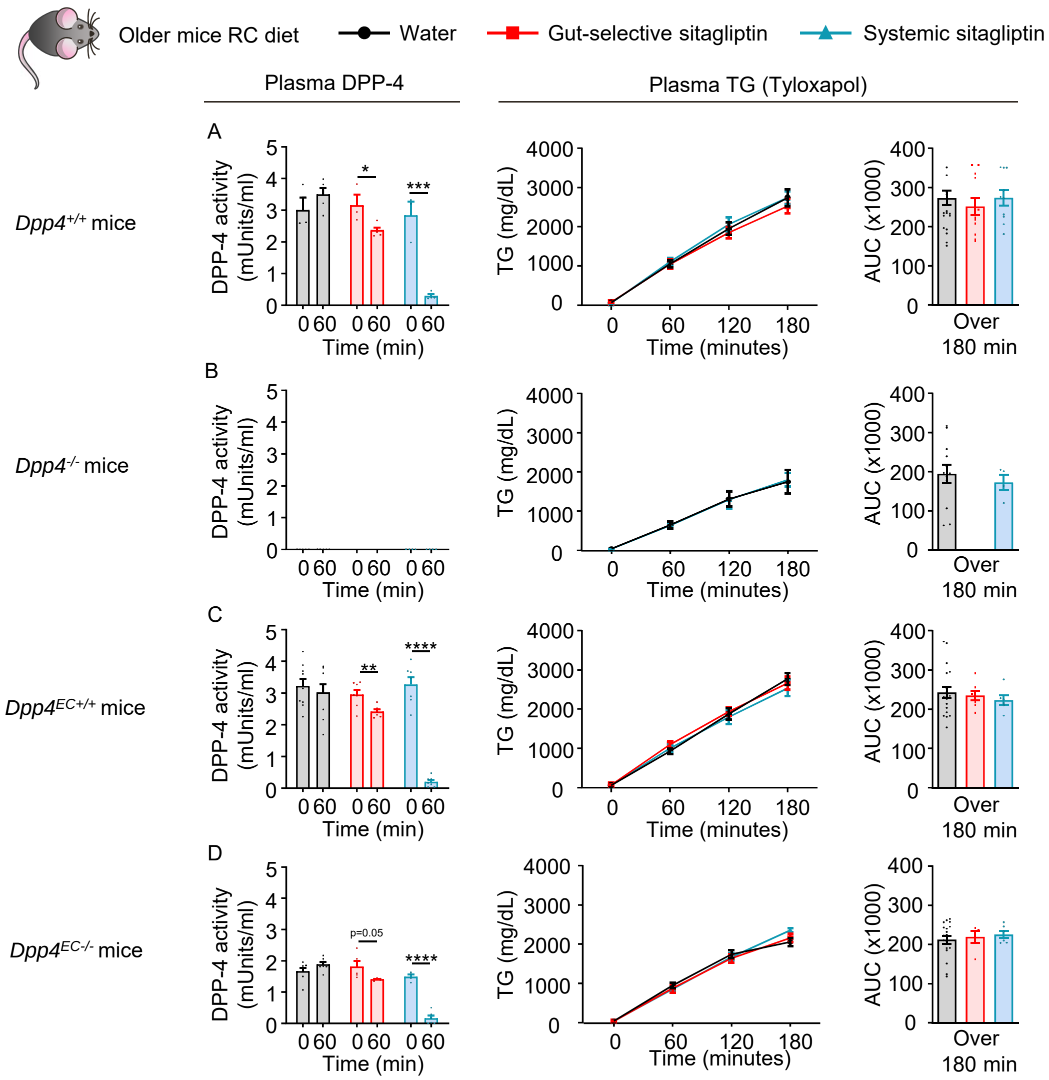

Supplemental Figure 5. Lipid tolerance in older RC-fed mice treated with sitagliptin. A-D: Plasma DPP-4 activity before and 60 minutes after olive oil gavage (200 µl) (left panels), and plasma TG and AUC over 180 minutes (right panels) in response to lipid and tyloxapol (0.5 g/kg) challenge and in response to water, gut-selective (14 µg/mouse) or systemic (10 mg/kg) dose of sitagliptin in 5-hour fasted 25-30-week-old *Dpp4*$^{+/+}$ (A, n=4-5/group for DPP-4, n=10-20 for TG), *Dpp4*$^{-/-}$ (B, n=4-5/group for DPP-4, n=5-12 for TG), *Dpp4*$^{EC+/+}$ (C, n=7-9/group for DPP-4, n=6-19 for TG), and *Dpp4*$^{EC-/-}$ (D, n=4-8/group for DPP-4, n=6-19 for TG) mice fed a regular chow (RC) diet. Data are presented as the means ± SEM. Each n represents a biological replicate from 2-4 independent cohorts of sex- and age-matched animals. *p<0.05, **p<0.01, ***p<0.001, ****p<0.0001 using Student's t-test.

Supplemental Figure 6

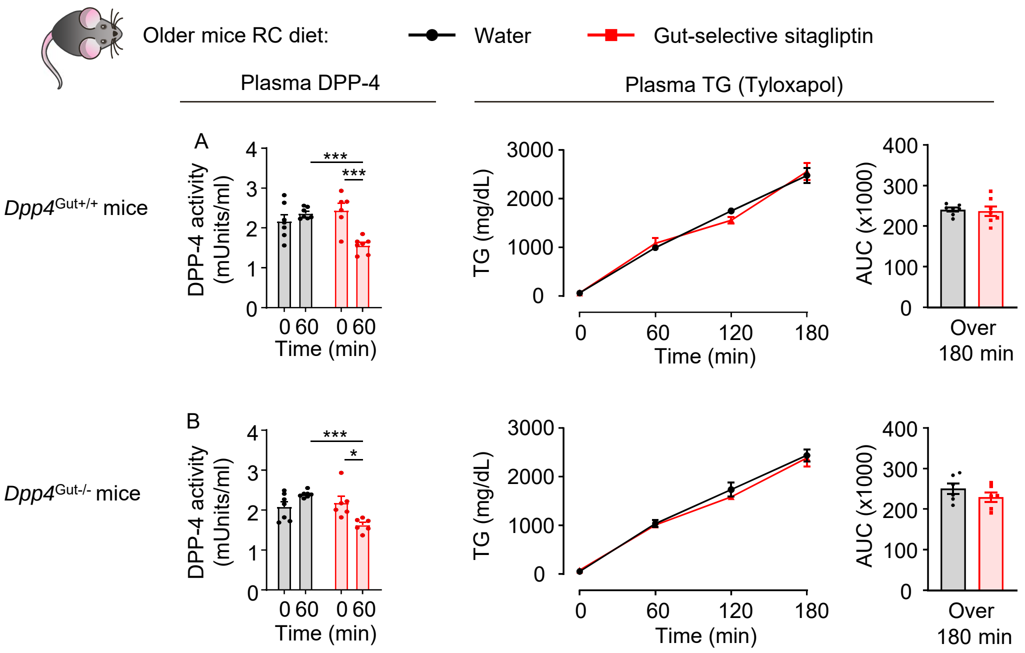

Supplemental Figure 6. Lipid tolerance in sitagliptin-treated *Dpp4*$^{\mathbf{Gut+/+}}$ and *Dpp4*$^{\mathbf{Gut-/-}}$ mice A-B: Plasma DPP-4 activity before and 60 minutes after olive oil gavage (200 µl) (left panels), and plasma TG and AUC over 180 minutes (right panels) in response to lipid and tyloxapol (0.5 g/kg) challenge and in response to water, or a gut-selective (14 µg/mouse) dose of sitagliptin in 5-hour fasted 25-30-week-old *Dpp4*$^{Gut+/+}$ (A, n=7/group), and *Dpp4*$^{Gut-/-}$ (B, n=6/group) mice fed a regular chow (RC) diet. Data are presented as the means ± SEM. Each n represents a biological replicate from 2 independent cohorts of sex- and age-matched animals. *p<0.05, ***p<0.001 using Student's t-test.

Supplemental Figure 7

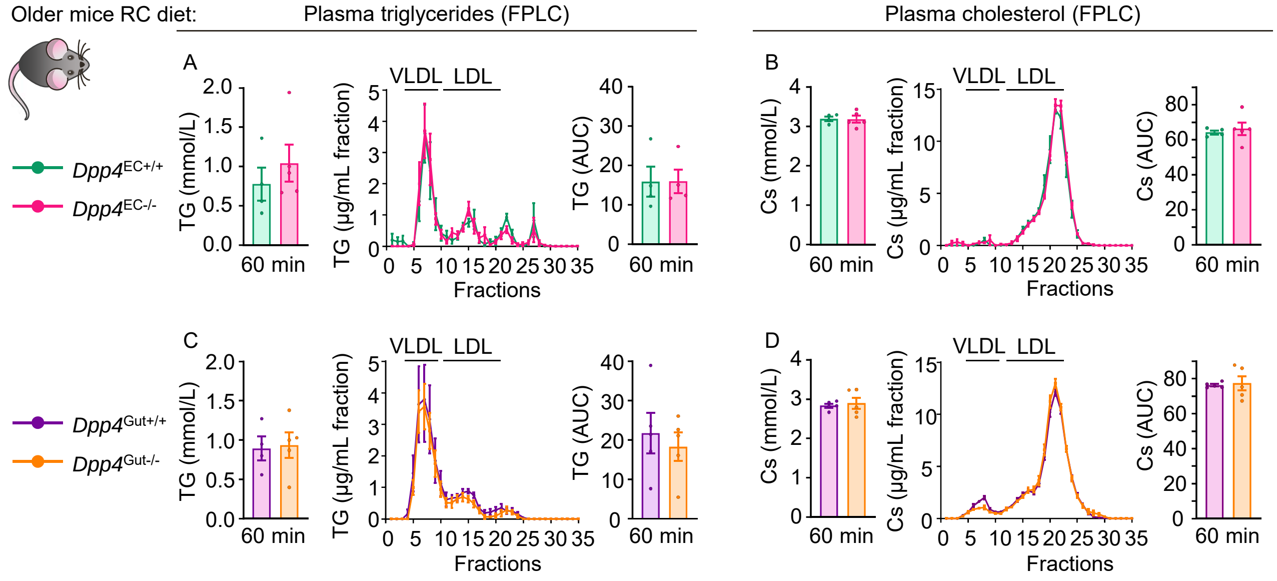

Supplemental Figure 7 (related to figure 10). FPLC lipoprotein profile is similar between genotypes in old chow-diet fed mice. **A-D:** Total (left panels) and FPLC profile and AUC (right panels) of triglycerides (TG) (A,C) and cholesterol (Cs) (B,D) in five-hour fasted 20-25-week-old *Dpp4*$^{EC-/-}$ vs *Dpp4*$^{EC+/+}$ (A-B, n=4-5/group), and *Dpp4*$^{Gut-/-}$ vs *Dpp4*$^{Gut+/+}$ (C-D, n=4-5/group) mice fed a regular chow (RC) diet, 60 minutes after olive oil gavage. Data are presented as the means ± SEM. Each n represents a biological replicate from one cohort each of sex- and age-matched animals.
